# Floral UV Features of Plant Species From a Neotropical Savanna

**DOI:** 10.3389/fpls.2021.618028

**Published:** 2021-05-07

**Authors:** Priscila Tunes, Maria Gabriela Gutierrez Camargo, Elza Guimarães

**Affiliations:** ^1^Laboratory of Ecology and Evolution of Plant-Animal Interactions, Postgraduate Program in Biological Sciences (Botany), Institute of Biosciences, São Paulo State University, Botucatu, Brazil; ^2^Phenology Laboratory, Department of Botany, Institute of Biosciences, São Paulo State University, Rio Claro, Brazil; ^3^Laboratory of Ecology and Evolution of Plant-Animal Interactions, Institute of Biosciences, São Paulo State University, Botucatu, Brazil

**Keywords:** biodiversity, floral guides, floral resource, flower colour, pollination systems, phylogeny, ultraviolet reflectance, UV-pattern

## Abstract

Despite the wide interest in flower colours, only after the end of the nineteenth-century studies started to comprise floral UV reflection, which is invisible to humans but visible to the major groups of pollinators. Many flowers and inflorescences display colour patterns, an important signal for pollinators, promoted by the presence of at least two different colours within flowers or inflorescences, including colours in the UV waveband. For Neotropical savanna plant species, we characterised floral UV features using UV-photography and reflectance measurements. We tested (i) whether floral UV features were constrained by their shared ancestry, (ii) whether floral UV features were associated with pollinators, and (iii) whether floral UV features were associated with floral traits mediating these interactions, including floral resource, type of attraction unit and presence/absence of non-UV colour patterns. Of 80 plant species, ca. 70% were UV-patternless, most of them UV-absorbing. Approximately 30% presented one of three types of UV-patterns: bullseye, contrasting corolla markings oriented toward floral resources or contrasting reproductive structures, which were all considered as floral guides. Floral UV features were phylogenetically constrained and were associated with pollinators, floral resources and attraction unit, but not with non-UV colour patterns. UV-patternless flowers were associated with most of the pollination systems, while UV-patterned flowers were mainly associated with bee-pollination. UV-absorbing flowers comprised the only category with hawkmoth- and butterfly-pollinated flowers, and a high percentage of hummingbird-pollinated species. Nocturnal pollinated species were also commonly UV-absorbing, except for one UV-reflecting bat-pollinated species and one beetle-pollinated species with UV-reflecting stigmas. All types of floral UV features were associated with nectar; however, flowers with contrasting reproductive structures were mainly associated with pollen. There was an association between UV-absorbing species and the presence of inflorescences and intermediate attraction units. Our results evince that phylogenetic relatedness can constraint floral UV features’ diversification, but combinations of evolutionary and ecological processes may be expected in this scenario.

## Introduction

Floral colour has always called humankind’s attention, and throughout time it was explored in several studies including genetic, ecological, and evolutionary approaches. However, until the end of the nineteenth century, these studies did not comprise the reflection of UV light, which was just recorded in flowers in 1891 by Knuth (1891). Ever since floral UV reflection has been attracting scientists’ interest as UV light is invisible to humans but visible to the major groups of pollinators. Indeed, floral UV-colouring can be perceived by many pollinators, such as bees (Chittka and Briscoe, 2001; Cronin et al., 2014), hummingbirds (Goldsmith and Goldsmith, 1979; Ödeen and Håstad, 2010), flies (Chittka and Briscoe, 2001; Cronin et al., 2014; Lunau, 2014), butterflies (Chittka and Briscoe, 2001; Cronin et al., 2014), beetles (Chittka and Briscoe, 2001), hawkmoths (White et al., 1994; Cronin et al., 2014), and bats (Müller et al., 2009), since all of these pollinator groups present photoreceptors that are sensitive to UV wavelengths. It is important to highlight that from a pollinator’s perspective, the UV wavelengths are only one of the wavebands perceived by animals that have UV photoreceptors (Kevan, 1979; Cronin et al., 2014). It acts together with the information from other photoreceptors that may perceive longer wavelengths, encompassing human-visible blue, green and red wavebands to create a colour (Chittka et al., 1994).

Floral colour diversity encompasses flowers that can be uniform in colour (non-patterned) and flowers that display colour patterns (patterned) (Koski, 2020). Floral colour patterns are formed by contrasting portions on the flowers, in the perianth or reproductive structures, which can be perceived by pollinators’ sensory systems (Chittka and Thomson, 2001). Floral UV-patterns, in the same way as human-visible patterns, are created by the local accumulation of pigments that can be UV-absorbing, such as flavonoids, including flavonols, chalcones and most anthocyanins (Thompson et al., 1972; Harborne, 1981; Kevan et al., 1996 and references therein; Schlangen et al., 2009), or UV-reflecting, such as carotenoids and some anthocyanins (Kevan et al., 1996 and references therein; Schlangen et al., 2009). Plant species that are more closely related in terms of phylogeny could show similar UV floral patterns (as shown for some clades of Rosaceae by Koski, 2020) since pigment biosynthesis and allocation can be genetically determined and regulated (Grotewold, 2006). Indeed, Camargo et al. (2019) showed that the amount of excitation caused by bee-pollinated flowers and hummingbird-pollinated flowers in their respective pollinator’s UV-photoreceptor was phylogenetically structured. However, still little is known about the evolutionary history of UV-patterns (Koski, 2020).

Although insect vision is limited to detect flower colour patterns at long-distance ranges (Hempel de Ibarra et al., 2015 and references therein), these patterns set important cues to discriminate among flowers at close range, to guide landing onto the flowers (Lunau, 1992; Hempel de Ibarra et al., 2015), and to locate resources within the flowers (Daumer, 1956; Dinkel and Lunau, 2001; Leonard and Papaj, 2011), thus, mediating plant-pollinator interactions (Medel et al., 2003; Koski and Ashman, 2014). Additionally, colour patterns can influence pollinator constancy and preferences (Hill et al., 1997; Bradshaw and Schemske, 2003; Horth et al., 2014), affecting the success of pollination (Johnson and Dafni, 1998; Goodale et al., 2014; Koski and Ashman, 2015). Furthermore, changes in UV features in flowers can even be associated with pollinator shifts (Bradshaw and Schemske, 2003; Sheehan et al., 2016; Martínez-Harms et al., 2020). Thus, it would be expected that the distribution of the types of floral UV features is shaped by pollinator preferences and behaviour through pollinator-mediated selection, as other floral traits (Jogesh et al., 2017).

Additionally, UV floral patterns can act as guides, used by pollinators to locate floral resources (Lunau, 1992; Koski and Ashman, 2014; Lunau et al., 2020). However, to our knowledge, possible associations between types of UV floral patterns and the types of floral resources presented by plant species; has not yet been tested. Nevertheless, pollinators might use flowers or inflorescences to make foraging decisions (Burdon et al., 2020), so that in some cases flowers act as attraction units and in other, inflorescences do (Ramirez et al., 1990). Thus, the pattern displayed by individual flower creates, together, a unique pattern that represents the inflorescence as a unit, such as a bullseye pattern, commonly displayed by Compositae inflorescences (e.g., Milne and Milne, 1958; Abrahamson and McCrea, 1977; Moyers et al., 2017). Consequently, there could be an association of the type of UV floral patterns presented by plants and their type of attraction unit.

More recently, the number of studies focused on understanding the distribution of floral UV patterns among flowering plants and on understanding the implications of such floral patterns to plant-pollinator interactions have increased substantially. However, there is still a wide range of questions to be answered regarding floral UV patterns. Here, we used plant species from a Neotropical savanna to characterise floral UV features and tested (i) whether floral UV features were constrained by their shared ancestry, (ii) whether floral UV features were related to pollinators, and (iii) whether floral UV features were associated with floral traits mediating these interactions, including floral resource, type of attraction unit and the presence/absence of non-UV colour patterns.

## Materials and Methods

### Study Sites

This study was conducted in natural populations of savanna physiognomies from “cerrado” vegetation, located in Botucatu (22°54’45” S, 48°30’13” W), Águas de Santa Bárbara (“Santa Bárbara Ecological Station,” 22°46’–22°41’S, 49°16’–49°10’W) municipalities, in São Paulo state, and São Roque de Minas municipality (“Serra da Canastra National Park,” 20°14’01” S, 46°26’40” W), in Minas Gerais state, Brazil. Sisgen authorisation #A90A83C and ICMBio/MMA/SISBIO authorisation #70131-1 for collection of biological samples. The “cerrado,” is a highly diverse Neotropical savanna vegetation (Pfadenhauer and Klötzli, 2020) in which flowering species are mainly pollinated by bees, but also by other vectors, such as hummingbirds, bats, flies, butterflies, beetles and moths (Gottsberger and Silberbauer-Gottsberger, 2006). To ensure that the proportion of pollination systems in our assemblage was representative of “cerrado” communities in general, we compared the observed ratios with those described by Gottsberger and Silberbauer-Gottsberger (2006) and Tunes et al. (2017) for other “cerrado” communities. For that, we used the Kruskall-Wallis test, after checking non-normality of the data. We found that the proportions of pollination systems in our study were similar to those recorded by Gottsberger and Silberbauer-Gottsberger (2006) and Tunes et al. (2017) (χ^2^ = 0.47043, *df* = 2, *p* = 0.7904; see the specific proportions in Supplementary Table 1), evincing the representativeness of our dataset.

### Assessment of Floral UV Features

We performed various expeditions to survey the UV features from flowers of savanna species. We conducted the field study throughout the years of 2019 to 2021, to capture the blooming period of a large number of plant species. To access the UV features, we used UV-photography, which allows us to see if and which UV patterns were present in flowers and inflorescences. We also validated the observed patterns with reflectance data of different floral parts. Based on these complementary data, UV-photographs, and reflectance data, we described floral UV features. Then, we classified the observed features into categories.

We photographed the UV reflectance of one to three flowers and inflorescences of 80 plant species (Supplementary Table 2) in studio conditions. For that, we used a hand-held UV light source, which emits light from 315 to 405 nm, with a peak at 365 nm, to illuminate the flowers. We excluded all human-visible light by using a camera with a modified sensor that only captures UV light from 340 to 400 nm, which corresponds exclusively to the UV portion of the light spectrum. In addition, this range corresponds to the spectral sensitivity of the UV-photoreceptors of a large variety of Hymenopteran pollinators (Chittka, 1992; Peitsch et al., 1992; Skorupski et al., 2007; Skorupski and Chittka, 2010), bird pollinators (Herrera et al., 2008; Ödeen and Håstad, 2010), and bat pollinators (Müller et al., 2009).

To collect the reflectance data, we used a spectrophotometer (Ocean Optics Jaz-EL200 UV-VIS) which collects reflectance data from 200 to 890 nm including the UV and human-visible wavelengths. We considered UV-reflecting when a given floral part reflects more than 5% between 300 and 400 nm, the UV-band. Although the mean reflectance of our data in the UV-band is around 3.5%, we opted by 5% to make our classification comparable with other community studies (e.g., Chittka et al., 1994; Camargo et al., 2019). As complementary information, we checked if the reflectance of a given floral structure presented marker points (Shrestha et al., 2013; Bukovac et al., 2017) located in the UV-band, that is, a stimulus promoted by rapid reflectance changes between 300 and 400 nm. We surveyed the marker points according to Camargo et al. (2019), using the “peakshape” function of the “pavo” package for R (Maia et al., 2019).

### Floral UV Features’ Phylogenetic Signal

We built a phylogenetic tree of the sampled plant species with PhyloMaker based on the “Phytophylo” megaphylogeny (Qian and Jin, 2016). We built the phylogenetic tree based on the third scenario, which creates polytomies by adding absent genera or species to their closest taxa. Then, we used δ (Borges et al., 2019) to verify the degree of phylogenetic signal between the presence of a given type of floral UV feature and the species’ phylogeny. This method calculates node entropy through a linear adaptation of Shannon entropy and then applies a Bayesian inferential scheme to calculate δ-value to verify the degree of phylogenetic signal in categorical traits (Borges et al., 2019). Higher gamma values indicate higher degrees of phylogenetic signal in the analysed categorical traits (Borges et al., 2019).

### Pollinators

Besides classifying the flower UV features, we also classified every plant species according to their pollinators, as described in the literature. When pollinators’ information was not available, we determined the most probable pollen vector based on flower attributes according to Faegri and Van der Pijl (1979) and Rosas-Guerrero et al. (2014). See Supplementary Table 2 for pollinator information for every plant species.

### Floral Resources and Attraction Units

We classified every plant species according to their floral resources, as described in the literature. If a plant species presented more than one type of floral resource, it was included in all the corresponding categories, since we cannot discriminate “*a priori*” which of the resources, present in each plant species, could be associated with its floral UV feature. We classified every plant species regarding their attraction unit, that is, single flowers, intermediate or inflorescences, based on the classification proposed by Ramirez et al. (1990). See Supplementary Table 2 for floral resource and attraction unit information for every plant species.

We classified every plant species according to the presence/absence of non-UV colour pattern according to the human colour vision, which is sensitive for wavelengths from 400 to 700 nm. The presence/absence of non-UV colour patterns was confirmed by the reflectance data collected as previously described. See Supplementary Table 2 for the presence/absence of non-UV colour patterns in every plant species.

### Statistical Analyses

We calculated phylogenetic signal (δ) based on the method proposed by Borges et al. (2019) for categorical variables. To evaluate if there was any association between the frequencies of each type of floral UV feature and pollinator group, between the frequencies of each type of floral UV feature and floral resource type, between the frequencies of each type of floral UV feature and attraction unit type, and between the frequencies of each type of floral UV feature and the presence/absence of non-UV colour patterns, we performed asymptotic generalised Pearson chi-squared tests with *post hoc* pairwise tests of independence. We carried out the statistical analyses in R v. 4.0.2 (R Core Team, 2020) with standard and additional packages: ape (Paradis and Schliep, 2019), circlise (Gu et al., 2014), chorddiag (Flor, 2020), coin (Hothorn et al., 2008), hrbrthemes (Rudis, 2020), patchwork (Pedersen, 2020), phytools (Revell, 2012), picante (Kembel et al., 2010), rcompanion (Mangiafico, 2020), tidyverse (Wickham et al., 2019), viridis (Garnier, 2018).

## Results

**FIGURE 1:**
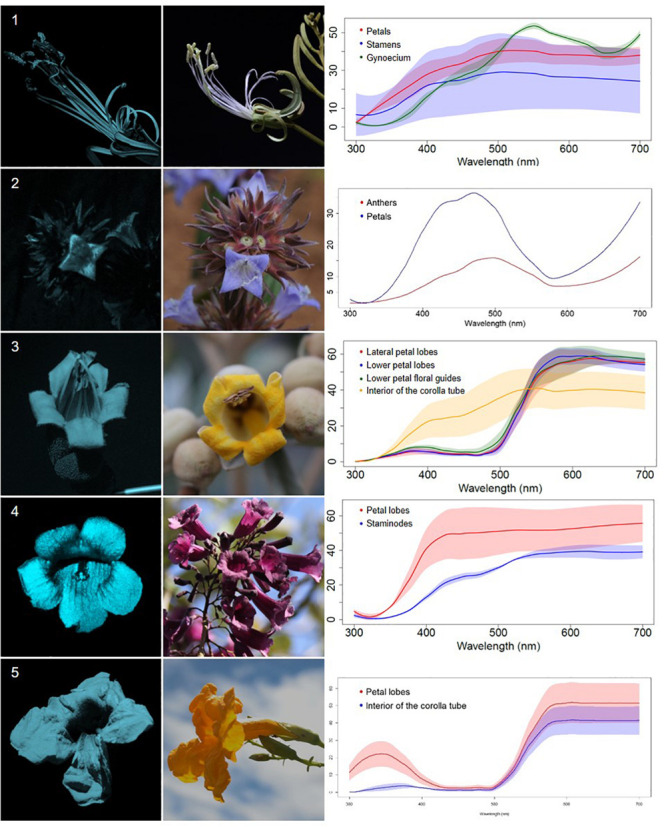
UV-reflecting non-patterned flowers (R) from a Neotropical savanna community. Each row comprises information of one species. First column: UV-photography. Second column: flower as seen by the human eye (conventional photography). Third column: reflectance curves of different portions of the flowers. Row 1. *Bauhinia rufa* (Bong.) Steud. (Leguminosae). Row 2. *Spermacoce poaya* A.St.-Hil. (Rubiaceae). Row 3. *Zeyheria montana* Mart. (Bignoniaceae). Row 4. *Jacaranda caroba* (Vell.) DC. (Bignoniaceae). Row 5. *Adenocalymma peregrinum* (Miers) L.G.Lohmann (Bignoniaceae).

**FIGURE 2:**
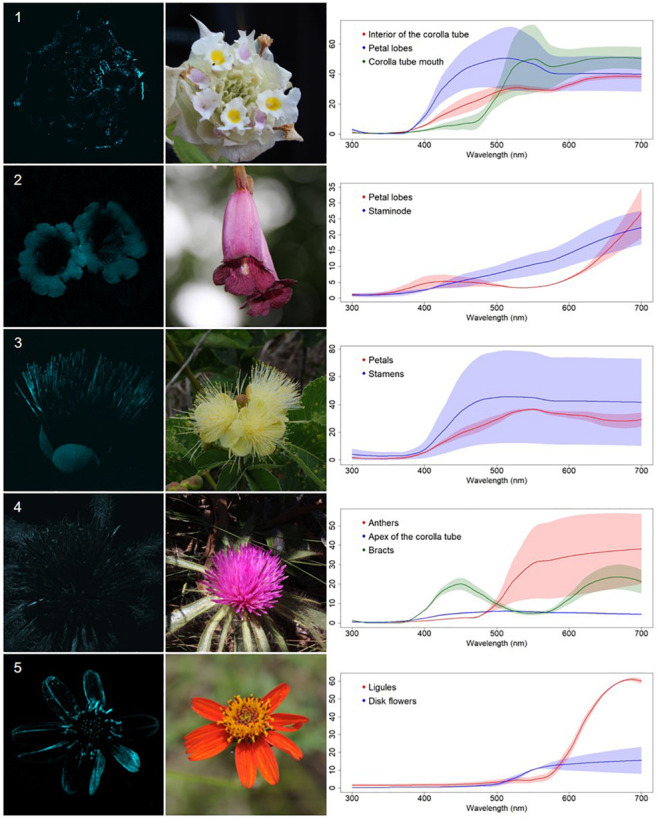
UV-absorbing non-patterned flowers (A) from a Neotropical savanna community. Each row comprises information of one species. First column: UV-photography. Second column: flower as seen by the human eye (conventional photography). Third column: reflectance curves of different portions of the flowers. Row 1. *Lippia lupulina* Cham. (Verbenaceae). Row 2. *Jacaranda rufa* Silva Manso (Bignoniaceae). Row 3. *Caryocar brasiliense* A.St.-Hil. (Caryocaraceae). Row 4. *Gomphrena macrocephala* A.St.-Hil. (Amaranthaceae). Row 5. *Bidens gardnerii* Baker (Compositae).

**FIGURE 3:**
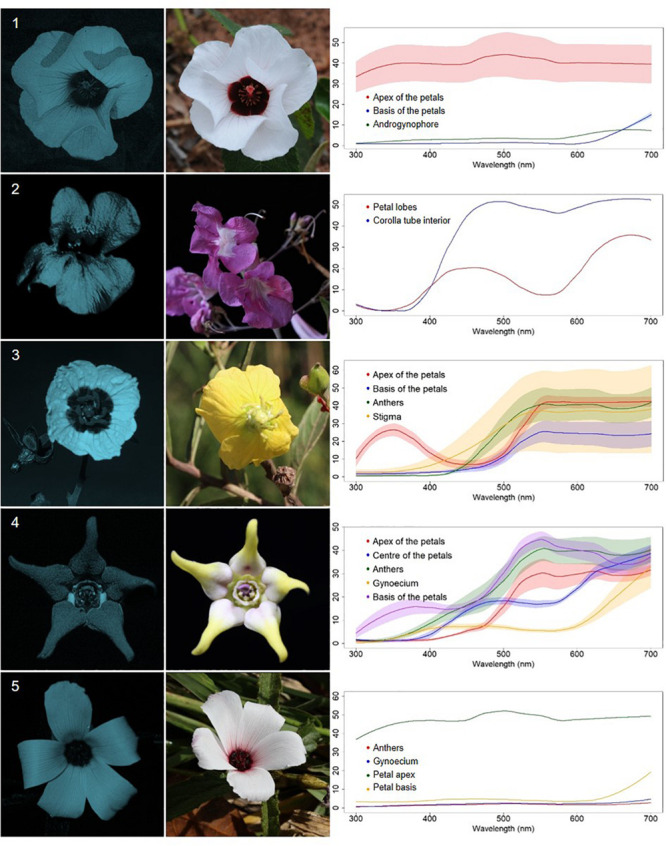
Bullseye UV-patterned flowers (BE) from a Neotropical savanna community. Each row comprises information of one species. First column: UV-photography. Second column: flower as seen by the human eye (conventional photography). Third column: reflectance curves of different portions of the flowers. Row 1. *Peltaea polymorpha* (A. St.-Hil.) Krapov. and Cristóbal (Malvaceae). Row 2. *Cuspidaria* sp. (Bignoniaceae). Row 3. *Ludwigia nervosa* (Poir.) H.Hara (Onagraceae). Row 4. *Oxypetalum appendiculatum* Mart. (Apocynaceae). Row 5. *Piriqueta aurea* (Cambess.) Urb. (Turneraceae).

**FIGURE 4:**
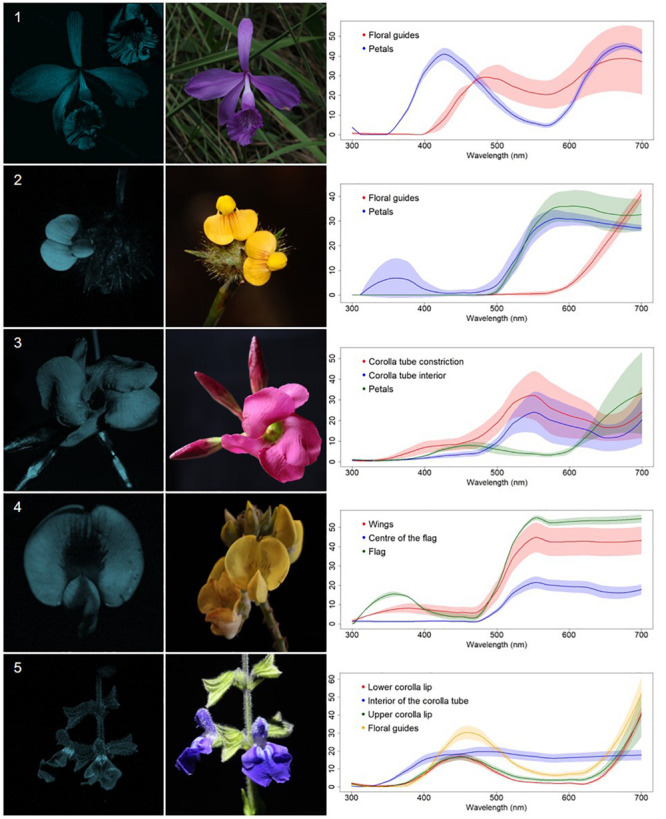
Contrasting corolla markings oriented towards floral resources UV-patterned flowers (CM) from a Neotropical savanna community. Each row comprises information of one species. First column: UV-photography. Second column: flower as seen by the human eye (conventional photography). Third column: reflectance curves of different portions of the flowers. Row 1. *Epistephium sclerophyllum* Lindl. (Orchidaceae). Row 2. *Stylosanthes guianensis* (Aubl.) Sw. (Leguminosae). Row 3. *Temnadenia violacea* (Vell.) Miers (Apocynaceae). Row 4. *Crotalaria micans* Link (Leguminosae). Row 5. *Salvia minarum* Briq. (Lamiaceae).

**FIGURE 5:**
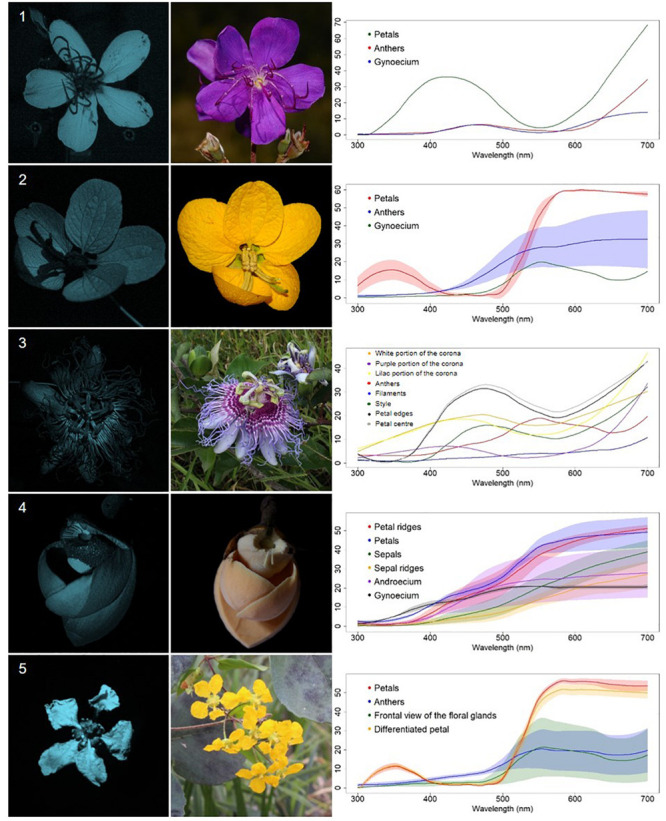
Contrasting reproductive structures UV-patterned flowers (CR) from a Neotropical savanna community. Each row comprises information of one species. First column: UV-photography. Second column: flower as seen by the human eye (conventional photography). Third column: reflectance curves of different portions of the flowers. Row 1. *Pleroma stenocarpa* (DC.) Cogn. (Melastomataceae). Row 2. *Senna rugosa* (G.Don) H.S.Irwin and Barneby(Leguminosae). Row 3. *Passiflora cincinnata* Mast. (Passifloraceae). Row 4. *Annona coriacea* Mart. (Annonaceae). Row 5. *Janusia guaranitica* (A.St.-Hil.) A.Juss. (Malpighiaceae).

**FIGURE 6:**
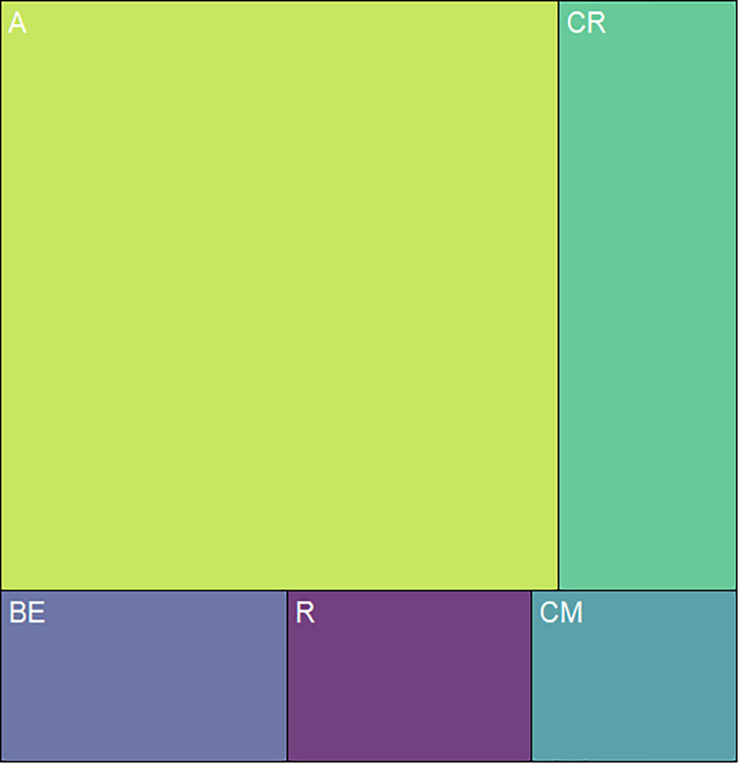
Distribution of the 80 sampled species among the types of UV features. The relative area occupied by each quadrilateral polygon corresponds to the relative occurrence of each floral UV category. From the sampled species, 7.5% presented flowers that belonged to the (R) category and 58.7% to the (A) category, which indicates that 66.2% of species were non-UV-patterned. The remaining 33.8% of species presented UV floral patterns, being that 8.75% presented (BE) pattern, 6.25% presented (CM) pattern and 18.75%, (CR) pattern. R, non-patterned UV-reflecting; A, non-patterned UV-absorbing; BE, bullseye; CM, contrasting corolla markings oriented towards floral resources; CR, contrasting reproductive structures.

We classified the 80 observed plant species, belonging to 68 genera, in 29 families, into five categories, based on floral UV features: (R) UV-reflecting; (A) UV-absorbing; (BE) bullseye; (CM) contrasting corolla markings oriented toward floral resources or reproductive structures (UV-reflecting markings in a flower with predominantly UV-absorbing corolla, and *vice versa*) and (CR) contrasting reproductive structures (UV-reflecting reproductive structures in a flower with UV-absorbing corolla, and *vice versa*) (Figures 1–5, Supplementary Figure 1, and Supplementary Table 2). It is noteworthy that 7.5% belonged to the (R) category and 58.75% to the (A) category, which indicates that 66.25% of species did not present any UV pattern but instead were homogeneously reflecting or absorbing UV (Figure 6). On the other hand, the remaining 33.75% of species presented UV floral patterns. We use the term UV-patterns here to refer to flowers that, differently from the two previous categories that were uniform in relation to UV properties, presented heterogeneous UV properties. These flowers showed a mixture of reflective and absorbing portions that creates various patterns within the attraction unit. These UV floral patterns could be considered as floral guides (*sensu* Dafni and Giurfa, 1999). From our sampled species, 8.75% presented (BE) pattern, 6.25% presented (CM) pattern and 18.75%, (CR) pattern (Figure 6).

**FIGURE 7:**
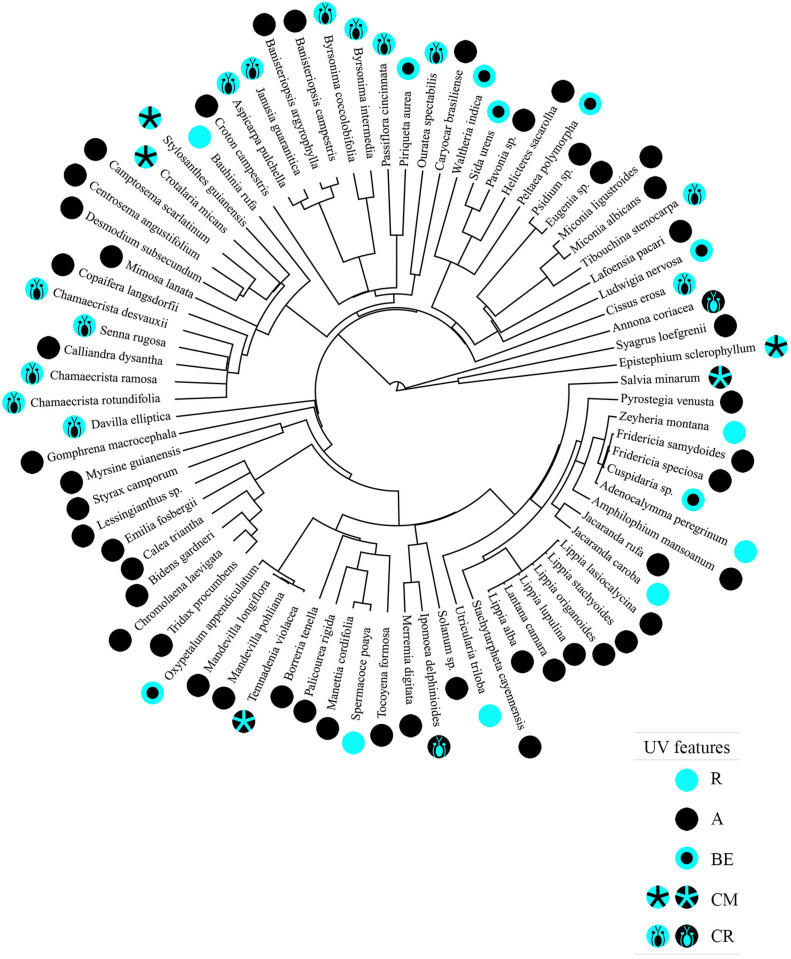
Phylogenetic tree representing 80 Neotropical savanna plant species based on the megaphylogeny by Qian and Jin (2016), including the floral UV features of each plant species. R, non-patterned UV-reflecting; A, non-patterned UV-absorbing; BE, bullseye; CM, contrasting corolla markings oriented toward floral resources; CR, contrasting reproductive structures. The types of floral UV features are represented by symbols in which blue represents UV-reflection and black represents UV-absorption. *Pleroma stenocarpa* and *Betencoutia scarlatina* are represented in the figure by their synonyms *Tibouchina stenocapa* and *Camptosema scarlatinum*, respectively, because the updated nomenclature was not compatible with the species name in the megaphylogeny.

We observed a significant degree of phylogenetic signal between the floral UV features and plant species phylogeny (δ = 1.731154; *p* < 0.001; Figure 7).

**FIGURE 8:**
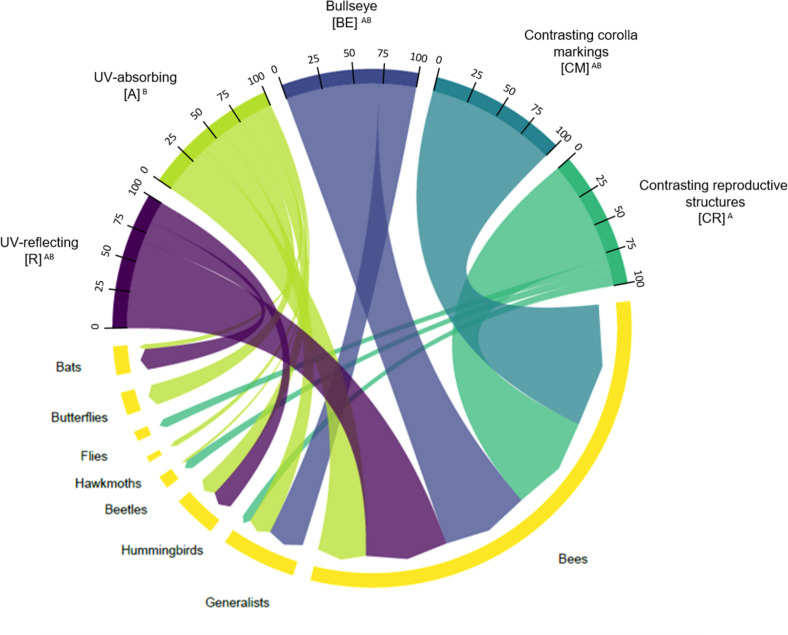
Chord diagram showing the relationship between the types of floral UV features and pollination systems of 80 plant species. The chords are unidirectional. Chord thickness corresponds to the percentage of species with each floral UV feature that is related to a given pollination system. Similar letters indicate that the UV categories were related to similar proportions of each pollination system (χ^2^ = 9.5294; *p* = 0.04915). See Supplementary Tables 3, 4 for percentages and detailed statistics. R, non-patterned UV-reflecting; A, non-patterned UV-absorbing; BE, bullseye; CM, contrasting corolla markings oriented towards floral resources; CR, contrasting reproductive structures.

We observed that there was an association between the type of floral UV feature and pollination systems (χ^2^ = 9.5294; *p* = 0.04915; Figure 8). See Supplementary Table 3 for percentages and detailed statistics. UV-reflecting flowers (R) were mainly associated with bee-pollination (66.6% of plant species), but also with hummingbird-, and bat-pollination. UV-absorbing flowers (A) were associated with almost all pollinator groups, except with fly-pollination and also presented high percentage of bee-pollinated species (40.4%). UV-absorbing flowers (A) were the only category associated with hawkmoth and butterfly-pollinated species (Figure 4). Regarding the plant species that presented floral UV-patterns, all three categories were mainly associated with bee-pollination. The species with a bullseye pattern (BE), besides being associated with bee-pollination (71.4%), were also associated with generalist pollination (28.6%) (Figure 4). It is noteworthy that flowers with contrasting markings in the corolla (CM) were exclusively associated with bee-pollination (Figure 4). Similarly, to the observed for (BE), (CR) species were associated primarily with bee-pollination (80.0%), but also with generalist pollination (6.6%) (Figure 8). (CR) was the only category that was associated with fly-pollination. See Supplementary Tables 3, 4 for percentages and detailed statistics.

**FIGURE 9:**
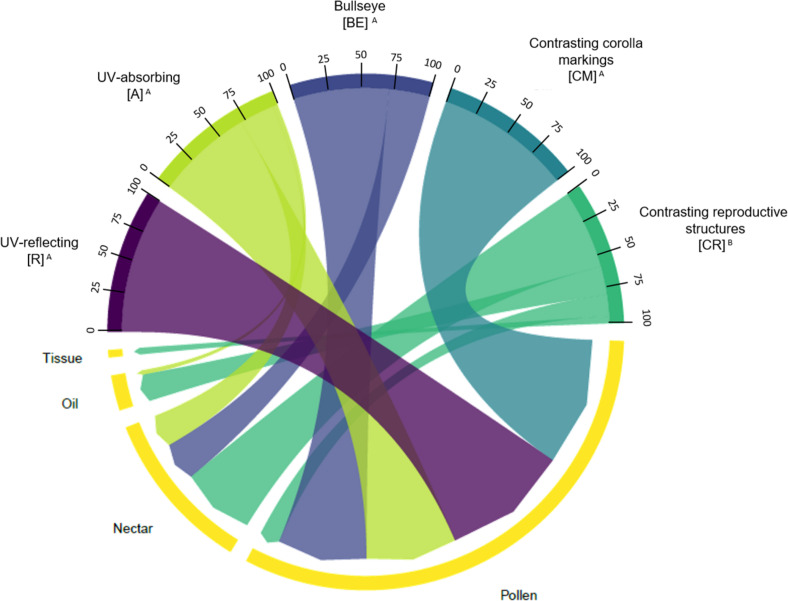
Chord diagram showing the relationship between the types of floral UV features and the type of floral resource of 80 plant species. The chords are unidirectional. Chord thickness corresponds to the percentage of species with each UV feature that is related to a given floral resource type. Similar letters indicate that the UV categories were related to similar proportions of each floral resource type (χ^2^ = 29.278; *p* = 6.862 × 10^– 6^). See Supplementary Tables 5, 6 for percentages and detailed statistics. R, non-patterned UV-reflecting; A, non-patterned UV-absorbing; BE, bullseye; CM, contrasting corolla markings oriented towards floral resources; CR, contrasting reproductive structures.

We found an association between floral UV features and floral resources (χ^2^ = 29.278; *p* < 0.0001; Figure 9). The most frequent floral resource observed was nectar, followed by pollen. (R), (A), (CM), and (BE) were similarly associated with high frequencies of plant species that have nectar as resource (from 70.0 to 100% of the plant species in each category). Indeed, (R) and (CM) had exclusively nectar as a resource. Species belonging to (BE) category also had pollen, and species from (A) category had also pollen and oil as resource (Figure 9). On the other hand, (CR) flowers differed from those categories and were associated primarily with pollen as resource (57.9%) and in a low frequency, with nectar (15.8%) (Figure 9). Additionally, (CR) species are the only ones to present tissue as a resource. See Supplementary Tables 5, 6 for percentages and detailed statistics.

**FIGURE 10:**
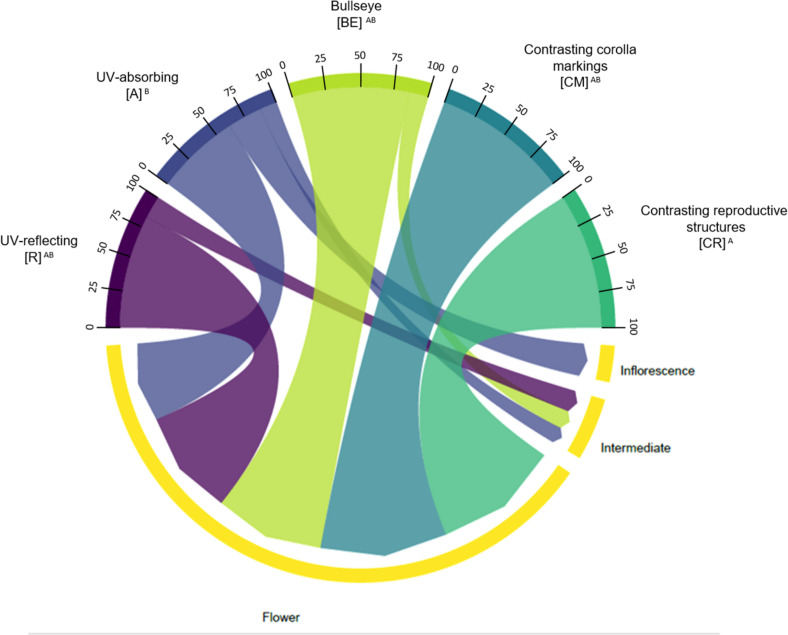
Chord diagram showing the relationship between the types of floral UV features and the type of attraction unit of 80 plant species. The chords are unidirectional. Chord thickness corresponds to the percentage of species with each UV feature that is related to a given attraction unit. Flowers within each UV category presented different percentages of species with each attraction unit type (χ^2^ = 12.33; *p* = 0.01506). See Supplementary Tables 7, 8 for percentages. R, non-patterned UV-reflecting; A, non-patterned UV-absorbing; BE, bullseye; CM, contrasting corolla markings oriented towards floral resources; CR, contrasting reproductive structures.

We also observed that there was an association between type of floral UV feature and type of attraction unit (χ^2^ = 12.33; *p* = 0.01506; Figure 10). There was an association between UV-absorbing species and the presence of inflorescences and intermediate attraction units. Additionally, (CR) and (CM) were only associated with flowers as attraction units. See Supplementary Tables 7, 8 for percentages and detailed statistics.

**FIGURE 11:**
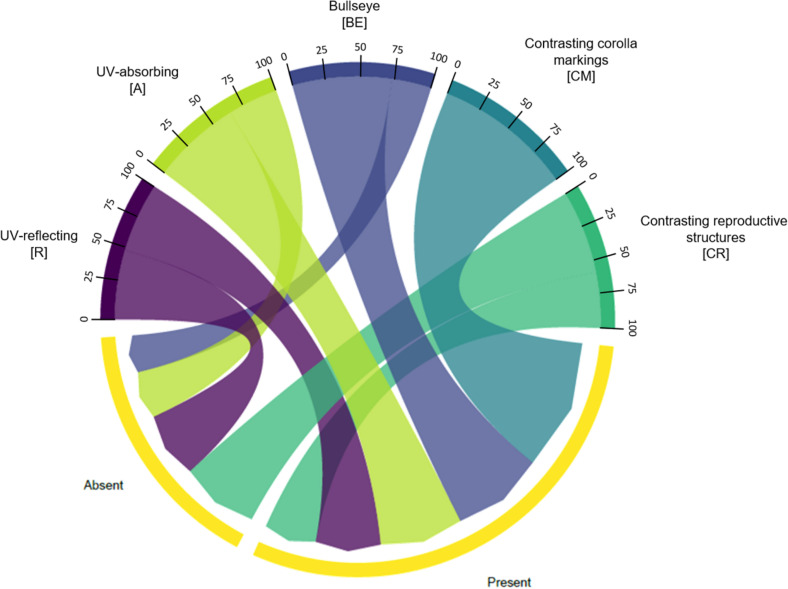
Chord diagram showing the relationship between the types of floral UV features and the presence/absence of non-UV colour patterns of 80 plant species. The chords are unidirectional. Chord thickness corresponds to the percentage of species with UV features that is related to a presence/absence of non-UV colour pattern. Flowers within each UV category presented similar percentages of species with presence/absence of non-UV colour patterns (χ^2^ = 6.7744; *p* = 0.1483). See Supplementary Table 9 for percentages. R, non-patterned UV-reflecting; A, non-patterned UV-absorbing; BE, bullseye; CM, contrasting corolla markings oriented towards floral resources; CR, contrasting reproductive structures.

We found no association between floral UV features and floral non-UV colour features (χ^2^ = 6.7744; *p* = 0.1483; Figure 11). See Supplementary Table 9 for percentages and detailed statistics.

## Discussion

We observed that most of the Neotropical savanna plant species are UV-patternless, belonging to (R) and (A) categories. However, 33.3% of the species show a composition of UV- reflecting and UV-absorbing areas that create different UV-patterns. We also show that floral UV features are influenced by plant species relatedness. We found association between the floral UV features presented by plant species and their pollinators, floral resources and the attraction units. However, there was no association of floral UV features with the presence/absence of non-UV colour patterns.

### Floral UV Features in Neotropical Savanna Plant Species

Besides being caused by floral nanoscale physical structures (Glover, 2014; Papiorek et al., 2014; Moyroud et al., 2017; van der Kooi et al., 2019), floral colour is a result of selective absorption or reflection of light caused by pigments (Kevan et al., 1996; van der Kooi et al., 2019). Thus, flowers can be homogeneously coloured or can present a combination of different colours, creating high contrast within the flower (Dafni and Kevan, 1996) and the enormous variety of patterns we observe in nature (Kevan et al., 1996; Schlangen et al., 2009). Despite the increase in contrasts created by colour patterns, our data shows a predominance of UV-patternless flowers, which means that almost 70% of species have pigments related to UV reflectance/absorbance uniformly distributed through the floral whorls (Glover, 2014).

On the other hand, 33.3% of the species presented UV-patterned flowers. We considered the three types of floral UV-patterns as “floral guides,” following Dafni and Giurfa (1999) definition, without discriminating the resource involved. Bullseye patterned flowers (BE) usually present an absorbing centre surrounded by a reflecting portion, which is ascribed to a spatial separation of flavonoids that matches exactly the UV-pattern (Brehm and Krell, 1975). Interestingly, the reproductive structures of all types of UV-patterned flowers are mostly located in UV-absorbing parts of the flowers. The species presenting contrasting marking pattern (CM) show both UV-absorbing or UV-reflecting markings converging at the region where the resource and reproductive structures are located. Also, most species showing contrasting reproductive structures (CR) have UV-absorbing androecium and gynoecium. In fact, UV-absorbing pigments, such as flavonoids, protect plants against UV-B radiation (Lee and Gould, 2002; Agati and Tattini, 2010; Landi et al., 2015). Anthers walls of some species may filter up to 98% of the UV-B radiation that reaches the pollen grains (Flint and Caldwell, 1983). So, UV-absorbing reproductive structures have been associated with pollen protection (Lunau, 2000 and references therein), since the high incidence of these wavelengths can decrease pollen production and viability (Demchik and Day, 1996; but see Peach et al., 2020). In general, flowers from savanna, a tropical open vegetation, are exposed to a high sunlight incidence (Pfadenhauer and Klötzli, 2020). So, UV-absorbing pigments in reproductive structures may represent an important trait for pollen protection, especially in the context of ozone layer degradation and UV incidence increase (Koski et al., 2020). In addition, the uniformly UV-absorbing corolla of the (A) category may provide extra protection, reducing the reflection of UV-light from petals to the anthers (Koski et al., 2020) and sheltering anthers since the bud stage (Flint and Caldwell, 1983).

Bees tend to make the first physical contact with a flower by means of an antennal reaction at an area of the flower displaying high chromatic contrast and high colour purity (Lunau et al., 1996; Rohde et al., 2013). Therefore, besides gamete protection, the UV-absorbing centre creates a centripetal increased gradient of chromatic contrast and colour purity, triggering bees’ preferences for colours of high spectral purity (Lunau, 1991a,b; Dafni and Giurfa, 1999). In addition to a marked contrast within the flower, floral UV-patterns may increase the contrast between the flower and the leaf-background, which usually absorbs ultraviolet light (Kevan and Backhaus, 1998; Camargo et al., 2014; van der Kooi et al., 2019).

### Floral UV Features’ Phylogenetic Signal

Flowers of closely related species from the Neotropical savanna presented similar floral UV features. Other studies also found phylogenetic restrictions related to UV-patterns (Koski and Ashman, 2016; Koski, 2020). In general, flower main colour is considered a labile floral trait, presenting weak or even no phylogenetic signal (McEwen and Vamosi, 2010; Shrestha et al., 2014; Muchhala et al., 2014; Bergamo et al., 2018). However, we must consider that flower colour patterns may be associated with the general flower structure (E-Vojtkó et al., 2020), such as resource location, reproductive structures position and exposure, which are probably more phylogenetically conservative traits. Indeed, we confirmed that floral UV features, although being an important signal for pollinators (Daumer, 1958; Koski and Ashman, 2014; Papiorek et al., 2016), present phylogenetic signal, which means that the phylogenetic relatedness of the plant species can help predict their UV-features.

### Floral UV Features and Plant-Pollinator Interaction

Evidence of floral colour and colour pattern importance for plant-pollinator interaction has been accumulated since the systematic observations by Sprengel (1793). In fact, in the present study, we observed a clear association between the type of floral UV feature and pollinator groups. This result could indicate that pollinators present preferences for specific types of floral UV features, which could lead to pollinators acting as selection agents upon this floral trait. Understanding the real role of pollinators in the angiosperm diversification process is a great challenge (van der Niet and Johnson, 2012). However, it is important to consider that the correlations of floral patterns with other floral traits, and their ecological role may also influence their phylogenetic distribution (Koski, 2020).

Here we show that both uniform floral UV categories, i.e., the UV-patternless flowers, are associated with an ample variety of pollination systems, being (A) associated with all pollinator groups, except flies. We must highlight that these flowers may present colour patterns related to human-visible colours, which are also important signals to different pollinators, such as bees, butterflies, and flies (Lunau et al., 1996; Kandori and Ohsaki, 1998; Heuschen et al., 2005; Hansen et al., 2012). Even though there are some simple connections between UV features and colours that are visible to humans, such as the fact that some UV-absorbing flavonoids also contribute to the yellow colour of Asteraceae and Leguminosae flowers, being called “yellow flavonols” (Harborne, 1967). However, carotenoids are also responsible for the flower’s yellow colour but are UV-reflecting (Kevan et al., 1996 and references therein; Schlangen et al., 2009). Therefore, making direct relations between human visible colours and UV-features is not as straightforward as it could seem, because the same colour in human vision can present different UV properties. Chittka et al. (1994), using a large sample size, showed that most flowers (∼74%) were UV-absorbing and the minority (∼26%) were UV-reflecting, regardless of what colour they displayed in the human-visible spectra. In the present study, we found a similar predominance of UV-absorbing flowers. Approximately 90% of the non-patterned flowers are UV-absorbing, while the remaining 10% are UV-reflective.

Even though flower’s colour contrast against the background is crucial for pollinators to locate a flower from a distance, floral colour patterns (floral guides), such as the ones presented by the (BE), (CM), and (CR) patterns, can act at short-distance pollinator orientation cues, mainly for insects (Manning, 1956; Lunau et al., 1996; Rohde et al., 2013). These patterns’ importance is ascribed to the fact that pollinators use them to discriminate among flowers from different species and to learn to obtain resources in a more accurate manner, which may represent less energy and time spent by pollinators (Kevan et al., 1996; Leonard and Papaj, 2011; Glover, 2014; de Jager et al., 2017), and higher plant fitness (Hansen et al., 2012). It is remarkable that in our study, these floral UV-patterns, (BE), (CM), and (CR) were associated almost exclusively with bee- and generalist-pollination, which for most plant species includes bees as pollinators (see Gottsberger and Silberbauer-Gottsberger, 2006). The external reflexive portion of the bullseye inflorescences may be used as a landing platform by pollinators, in contrast, the UV-absorbing centre of the inflorescence acts as a floral guide (Daumer, 1956, 1958). Such floral guide is perceived by bees, which show probing responses at the boundary between UV-reflecting and UV-absorbing floral regions (Daumer, 1958; Burkhardt et al., 1967; Papiorek et al., 2016). In fact, studies show that UV-patterned flowers may be more conspicuous to insects than non-patterned (Koski and Ashman, 2014; Papiorek et al., 2016) and that the presence of floral patterns may increase bees’ floral constancy (Scora, 1964). Bees can also learn which floral pattern is more profitable and change visiting behaviour (Xu and Plowright, 2017). So, it has been shown in several ways that floral UV-patterns can also influence pollinator choice and behaviour (Peterson et al., 2015; Brock et al., 2016; Papiorek et al., 2016; Koski, 2020), like colour patterns in human-visible wavelengths.

All the hummingbird-pollinated species in our study were UV-patternless, being most of them UV-absorbing. Our results are consistent with other studies that show that patterned flowers are less common in hummingbird-pollinated species than in bee-pollinated ones (Proctor and Yeo, 1973; Faegri and Van der Pijl, 1979; Camargo et al., 2019). Additionally, the absence of floral guides, coupled with UV-absorbing red flowers is seen as achromatic by bees, which creates a private niche for hummingbirds to explore these flowers without competing with bees for floral resources (Lunau et al., 2011; Papiorek et al., 2013; Camargo et al., 2019). This can be observed in 87.5% of the hummingbird-pollinated species in our study. The only species UV-reflecting (R) do not present any colour trait specifically related to hummingbird-pollination.

It is noteworthy that most nocturnal insect-pollinated species in our study are UV-absorbing (A). These species were pollinated by hawkmoths and beetles, which present colour vision even in moonless nights (Johnsen et al., 2006; Goyret et al., 2008; Théry et al., 2008) and eyes with high light sensitivity but low spatial resolution (Hempel de Ibarra et al., 2015). They also show slow image processing, which can affect insect behaviour, especially regarding flight speed and trajectory (Sponberg et al., 2015). In this context, one could expect that the presence of floral colour patterns, regardless of the wavelength, would be hard to distinguish by nocturnal insect pollinators. However, there is evidence that hawkmoths can perceive UV bullseye patterns in dim light (Hirota et al., 2019). Bats use different sensory cues for perceiving the surrounding environment and may constantly integrate information obtained through echolocation and vision (Boonman et al., 2013). They have S opsin genes that are sensitive to UV light, enabling them to use this colour especially at dawn and at dusk, when UV light is relatively more abundant (Wang et al., 2004). There is growing evidence of the general UV-sensitivity in bats and Gorresen et al. (2015) bring solid corroboration of widespread dim-light UV vision in bats. So, the corolla and androecium UV-reflecting (R) in *Bauhinia rufa*, a bat-pollinated species, may represent important cues for flower location by this pollinator group.

The sampled butterfly-pollinated species in our study were non-patterned, UV-absorbing (A) and most of them presented patterns in the human-visible wavelengths. Even though butterflies are mainly associated with patterned flowers (Proctor and Yeo, 1973; Kandori and Ohsaki, 1998), they can perceive flowers in nature regardless of the presence of UV patterns or uniform UV reflectance. Especially because butterflies present a high capability to discriminate colours from UV to deep red (Stavenga and Arikawa, 2006). On the other hand, even with an unclear colour preference, flies are usually related to yellowish flowers (Lunau, 2014), such as the (EC) fly-pollinated species registered in our study. The absence of UV reflectance does not discourage fly-pollination of the tropical *Hypoxis camerooniana* (Klomberg et al., 2019) but the presence of UV pattern favoured fly visitation of the temperate *Argentina anserina* flowers. Flies are sensitive to UV (Chittka and Briscoe, 2001; Cronin et al., 2014; Lunau, 2014) and maybe tuned to yellow UV-patterned flowers (An et al., 2018), similar to *Cissus erosa*, the fly-pollinated species sampled here.

In the present study, we verified that there is an association between the type of floral UV feature and floral resources. Both UV-patternless categories, (R) and (A), and two patterned categories, (BE) and (CM), are mostly associated with nectar. Nectar is the most common trophic resource exploited by virtually all pollinator groups (Nepi, 2017; Nepi et al., 2018). The separation observed here shows some connections among floral UV features, the type of floral resource and the pollinator group that exploit it, since butterflies, hawkmoths, bats, and hummingbirds are associated with non-patterned flowers, (R) and (A), which, in turn, present mainly nectar as a resource. (BE) and (CM) are mainly associated with bees and other insects that also search for nectar. The (CM) category presented a mixture of species, with some reflecting and others absorbing UV at the lines, showing UV-absorbing lines that can act as an honest signal (Pélabon et al., 2012; Lunau et al., 2020). (CR) species differ from all the other categories, by being mainly associated with pollen as floral resource, which is exploited by social and solitary bees for adult and larval provisions (Cane and Tepedino, 2017). Additionally, this floral UV category could be related with resource signalling, since yellow and UV-absorbing pollen and anthers (or mimics) trigger behavioural responses in bees and flies (Lunau et al., 2017 and references therein). Interestingly, oil-collecting bees are sensitive to floral visual changes (Ferreira and Torezan-Silingardi, 2013; Melo et al., 2018) and half of the Malpighiaceae species (mainly having oil as resource) show UV-absorbing reproductive structures (CR), a pattern usually associated with pollen-flowers (Lunau et al., 2017).

Individual flowers can present all the types of UV-features considered here, but inflorescences were always UV-absorbing. To our knowledge, this is the first study that analyses the relationship between colour patterns and the unit of attraction and further studies are necessary considering these arrangements in experiments testing for pollinators’ perception. Although we found no association between the presence/absence of non-UV colour patterns and UV features, most of the inflorescences that were UV-absorbing showed colour patterns in other wavebands. These patterns could be created within a flower or among flowers within inflorescences, like observed here for many Verbenaceae species.

In general, it has been shown that conspicuous flowers can receive more pollinator visits (Chittka et al., 2001), which might represent selection pressures toward more contrasting and distinguished visual features. Indeed, floral UV-patterns may favour bee attraction (Rae and Vamosi, 2013; Klomberg et al., 2019) and may increase the probability of the pollinator reaching floral resources, reducing handling time (Dinkel and Lunau, 2001; Leonard and Papaj, 2011). In spite of the predominance of UV-patternless flowers in butterfly-, hummingbird-, beetle-, bat-, and hawkmoth-pollinated species, we cannot disregard the importance of UV patterns for these pollinators until there are experimental evidences. Additionally, we confirmed that floral UV features, although being reportedly important signal for pollinators, are phylogenetically constrained. Therefore, the distribution of floral UV-features in nature cannot be ascribed to a single ecological or evolutionary factor. Thus, we still need to study wider assemblages of plant species, with different pollination systems to test some evolutionary hypotheses and to gain a broader knowledge of the selective pressures operating on UV features. In this context, adding data from a globally widespread biome, such as savanna, can bring a deeper understanding of ecological and evolutionary processes involved in floral UV feature diversification.

## Data Availability Statement

The original contributions presented in the study are included in the article/Supplementary Material, further inquiries can be directed to the corresponding author.

## Author Contributions

EG and PT conceptualised and designed the study. PT organised the database and performed the statistical analysis. EG, PT, and MC wrote the first draft of the manuscript. All authors contributed to manuscript revision, read, and approved the submitted version.

## Conflict of Interest

The authors declare that the research was conducted in the absence of any commercial or financial relationships that could be construed as a potential conflict of interest. The reviewer KL declared a past co-authorship with one of the authors MC to the handling Editor.
