# Supplementary material for: Floral UV Features of Plant Species From a Neotropical Savanna
Source: Front Plant Sci. 2021 May 7;12:618028. doi: 10.3389/fpls.2021.618028 (PMC8137824; doi:10.3389/fpls.2021.618028)
Supplement: Supplementary file 1 [file Data_Sheet_1.pdf]

Figure 1. Flower colour features of plant species from a Neotropical savanna community. Each line comprises information of one species. First column: UV-photography. Second column: flower as seen by the human eye (conventional photography). Third column: reflectance curves of different portions of the flowers. UV-reflecting non-patterned flowers [R]: Row 1. *Utricularia triloba* Benj. (Lentibulariaceae). UV-absorbing nonpatterned flowers [A]: Row 2. *Tocoyena formosa* (Cham. & Schltdl.) K.Schum. (Rubiaceae). Row 3. *Mandevilla pohliana* (Stadelm.) A.H.Gentry (Apocynaceae). Row 4. *Lantana camara* L. (Verbenaceae). Row 5. *Emilia fosbergii* Nicolson (Compositae). Row 6. *Miconia ligustroides* (DC.) Naudin (Melastomataceae). Row 7. *Pyrostegia venusta* (Ker Gawl.) Miers (Bignoniaceae). Row 8. *Tridax procumbens* (L.) L. (Compositae). Row 9. *Palicourea rigida* Kunth (Rubiaceae). Row 10. *Calea triantha* (Vell.) Pruski (Compositae). Row 11. *Amphilophium mansoanum* (DC.) L.G.Lohmann (Bignoniaceae). Row 12. *Merremia digitata* (Spreng.) Hallier (Convolvulaceae). Row 13. *Lippia lasiocalycina* Cham. (Verbenaceae). Row 14. *Lippia alba* (Mill.) N.E.Br. ex Britton & P.Wilson (Verbenaceae). Row 15. *Calliandra dysantha* Benth. (Leguminosae). Row 16. *Psidium* sp. (Myrtaceae). Row 17. *Fridericia speciosa* Mart. (Bignoniaceae). Row 18. *Lippia origanoides* Kunth (Verbenaceae). Row 19. *Lafoensia pacari* A.St.-Hil. (Lythraceae). Row 20. *Manettia cordifolia* Mart. (Rubiaceae). Row 21. *Styrax camporum* Pohl (Styracaceae). Row 22. *Mandevilla longiflora* (Desf.) Pichon (Apocynaceae). Row 23. *Desmodium subsecundum* Vogel (Leguminosae). Row 24. *Fridericia samydoides* (Cham.) L.G.Lohmann (Bignoniaceae). Row 25. *Betencourtia scarlatina* (Mart. ex Benth,) L.P.Queiroz (Leguminosae). Row 26. *Helicteres sacarolha* A.St.-Hil., Juss. & Cambess. (Malvaceae). Row 27. *Pavonia* sp. (Malvaceae). Row 28. *Centrosema angustifolium* (Kunth) Benth. (Leguminosae). Row 29. *Copaifera langsdorfii* Desf. (Leguminosae). Row 30. *Eugenia* sp. (Myrtaceae). Row 31. *Mimosa lanata* Benth. (Leguminosae). Row 32. *Miconia albicans* (Sw.) Triana (Melastomataceae). Row 33. *Stachytarpheta cayennensis* (Rich.) Vahl (Verbenaceae). Row 34. *Borreria tenella* (Kunth) Cham. & Schltdl. (Rubiaceae). Row 35. *Lessingianthus* sp. (Compositae). Row 36. *Banisteriopsis campestris* (A.Juss.) Little (Malpighiaceae). Row 37. *Croton campestris* A.St.-Hil. (Euphorbiaceae). Row 38. *Solanum* sp. (Solanaceae). Row 39. *Myrsine guianensis* (Aubl.) Kuntze (Myrsinaceae). Row 40. *Lippia stachyoides* Cham. (Verbenaceae). Row 41. *Syagrus loefgrenii* Glassman (Arecaceae). Row 42. *Banisteriopsis argyrophylla* (A.Juss.) B.Gates. Row 43. *Chromolaena laevigata* (Lam.) R.M.King & H.Rob. (Compositae). Bullseye UV-patterned flowers [BE]: Row 44. *Sida* cf. urens (Malvaceae). Row 45. *Waltheria indica* L. (Malvaceae). Contrasting reproductive structures UV-patterned flowers [CR]: Row 46. *Byrsonima coccolobifolia* Kunth (Malpighiaceae). Row 47. *Byrsonima intermedia* A.Juss. (Malpighiaceae). Row 48. *Ipomoea delphinioides* Choisy (Convolvulaceae). Row 49. *Chamaecrista rotundifolia* (Pers.) Greene (Leguminosae). Row 50. *Ouratea spectabilis* (Mart. ex Engl.) Engl. (Ochnaceae). Row 51. *Chamaecrista desvauxii* var. desvauxii (Leguminosae). Row 52. *Chamaecrista ramosa* (Vogel) H.S.Irwin & Barneby (Leguminosae). Row 53. *Davilla elliptica* A.St.-Hil. (Dilleniaceae). Row 54. *Aspicarpa pulchella* (Griseb.) O'Donell & Lourteig (Malpighiaceae). Row 55. *Cissus erosa* Rich. (Vitaceae).

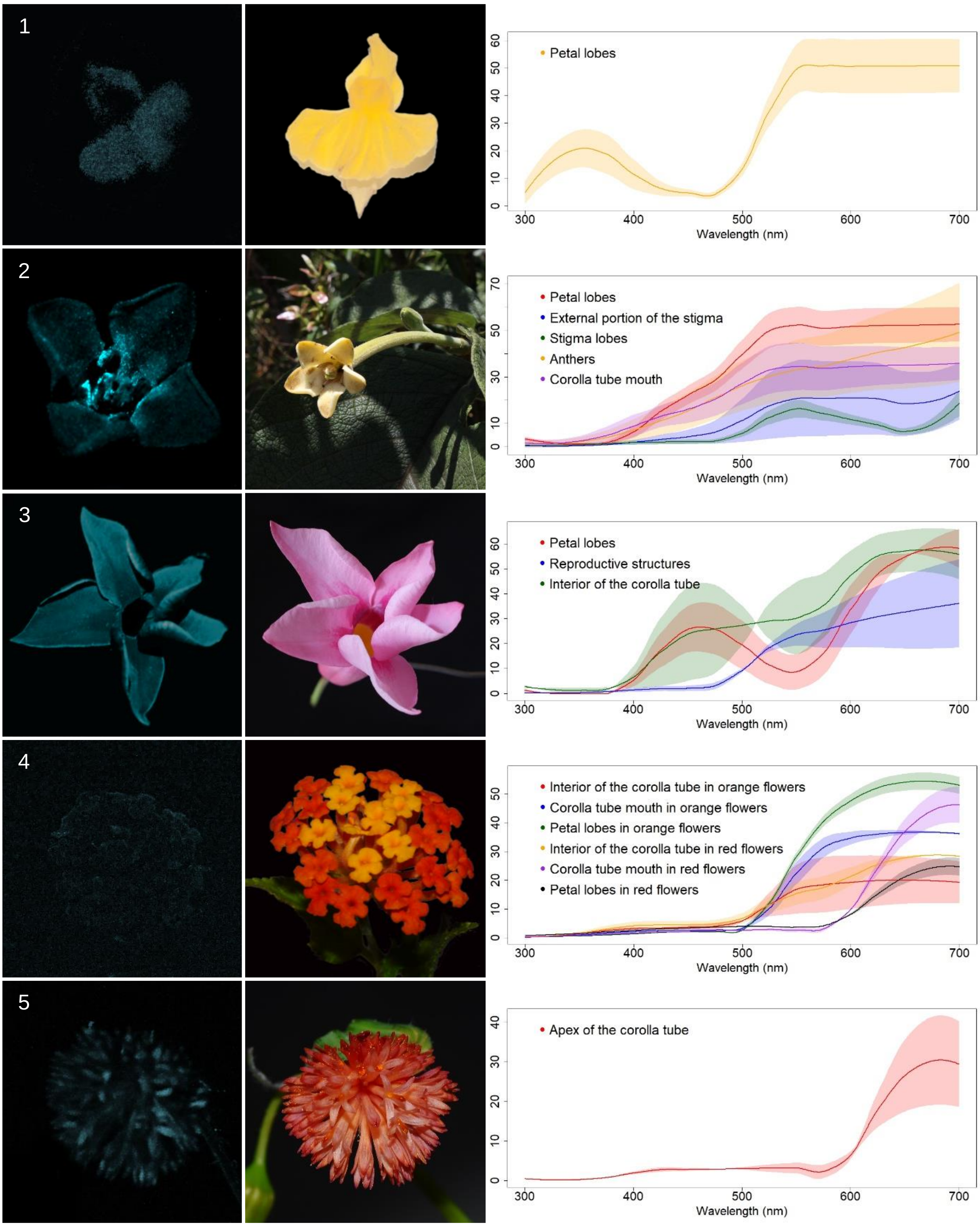
1
Petal lobes
Wavelength (nm)
2
Petal lobes
External portion of the stigma
Stigma lobes
Anthers
Corolla tube mouth
Wavelength (nm)
3
Petal lobes
Reproductive structures
Interior of the corolla tube
Wavelength (nm)
4
Interior of the corolla tube in orange flowers
Corolla tube mouth in orange flowers
Petal lobes in orange flowers
Interior of the corolla tube in red flowers
Corolla tube mouth in red flowers
Petal lobes in red flowers
Wavelength (nm)
5
Apex of the corolla tube
Wavelength (nm)

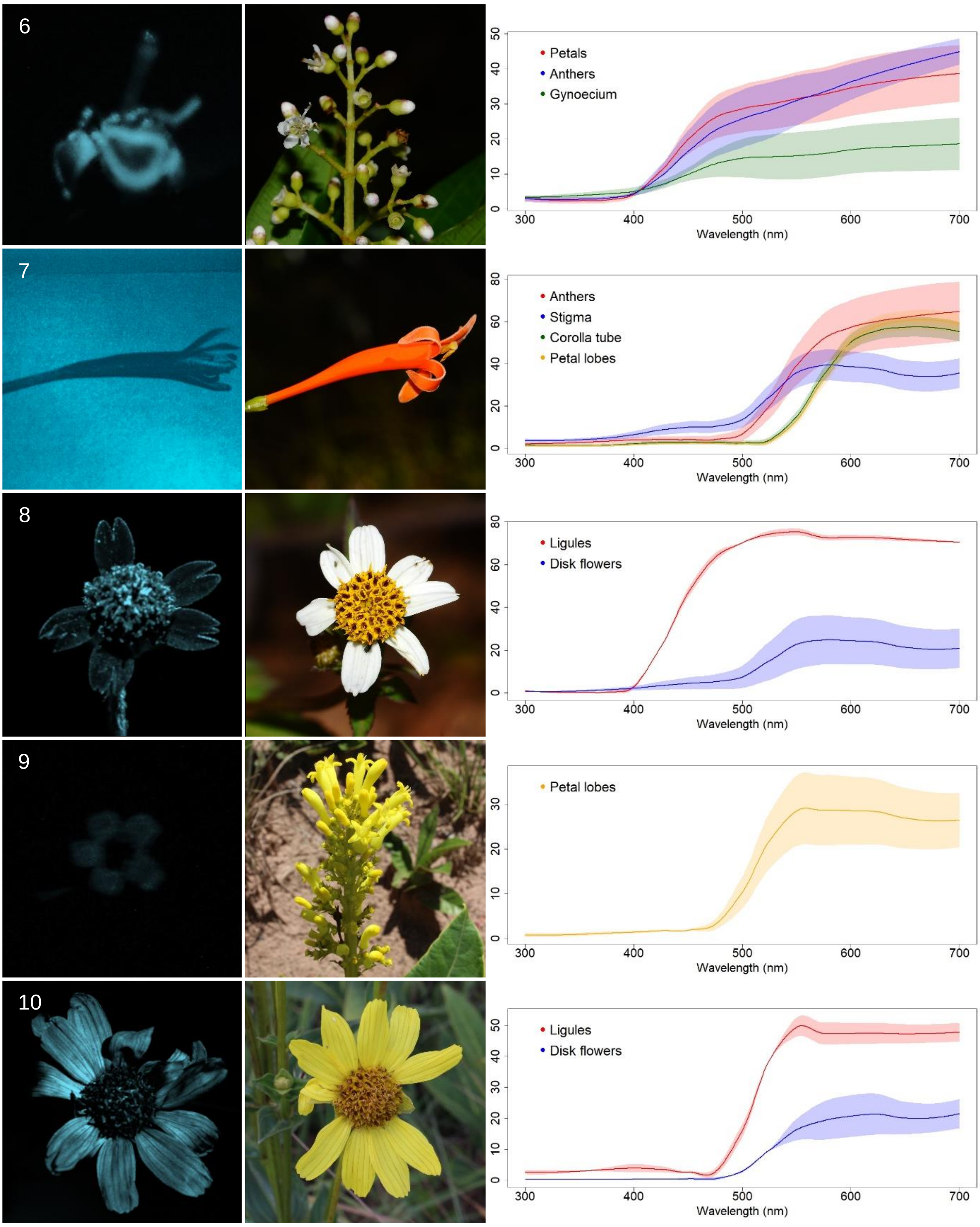
6
Petals
Anthers
Gynoecium
0 10 20 30 40 50
300 400 500 600 700
Wavelength (nm)
7
Anthers
Stigma
Corolla tube
Petal lobes
0 20 40 60 80
300 400 500 600 700
Wavelength (nm)
8
Ligules
Disk flowers
0 20 40 60 80
300 400 500 600 700
Wavelength (nm)
9
Petal lobes
0 10 20 30
300 400 500 600 700
Wavelength (nm)
10
Ligules
Disk flowers
0 10 20 30 40 50
300 400 500 600 700
Wavelength (nm)

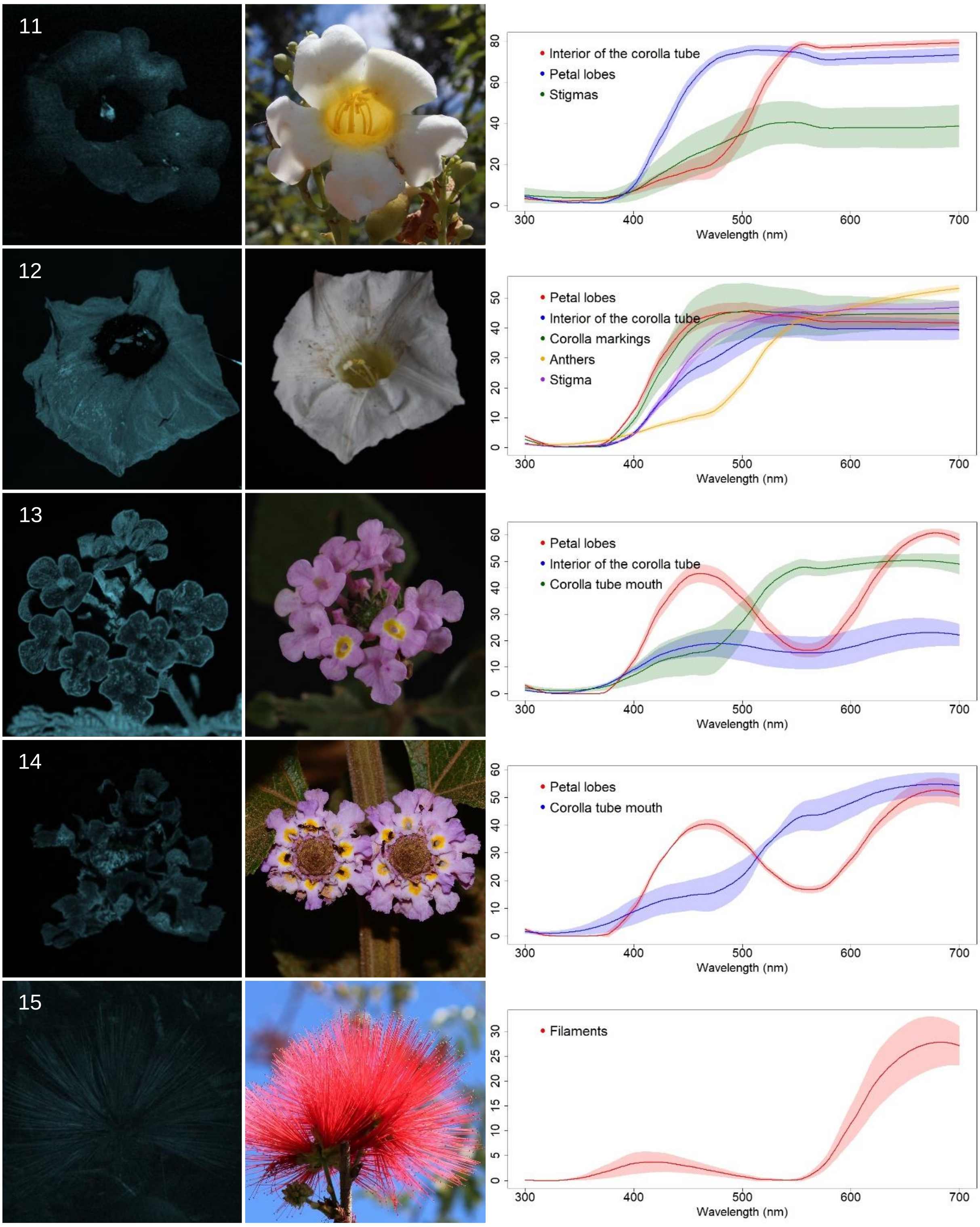
11
Interior of the corolla tube
Petal lobes
Stigmas
Wavelength (nm)
12
Petal lobes
Interior of the corolla tube
Corolla markings
Anthers
Stigma
Wavelength (nm)
13
Petal lobes
Interior of the corolla tube
Corolla tube mouth
Wavelength (nm)
14
Petal lobes
Corolla tube mouth
Wavelength (nm)
15
Filaments
Wavelength (nm)

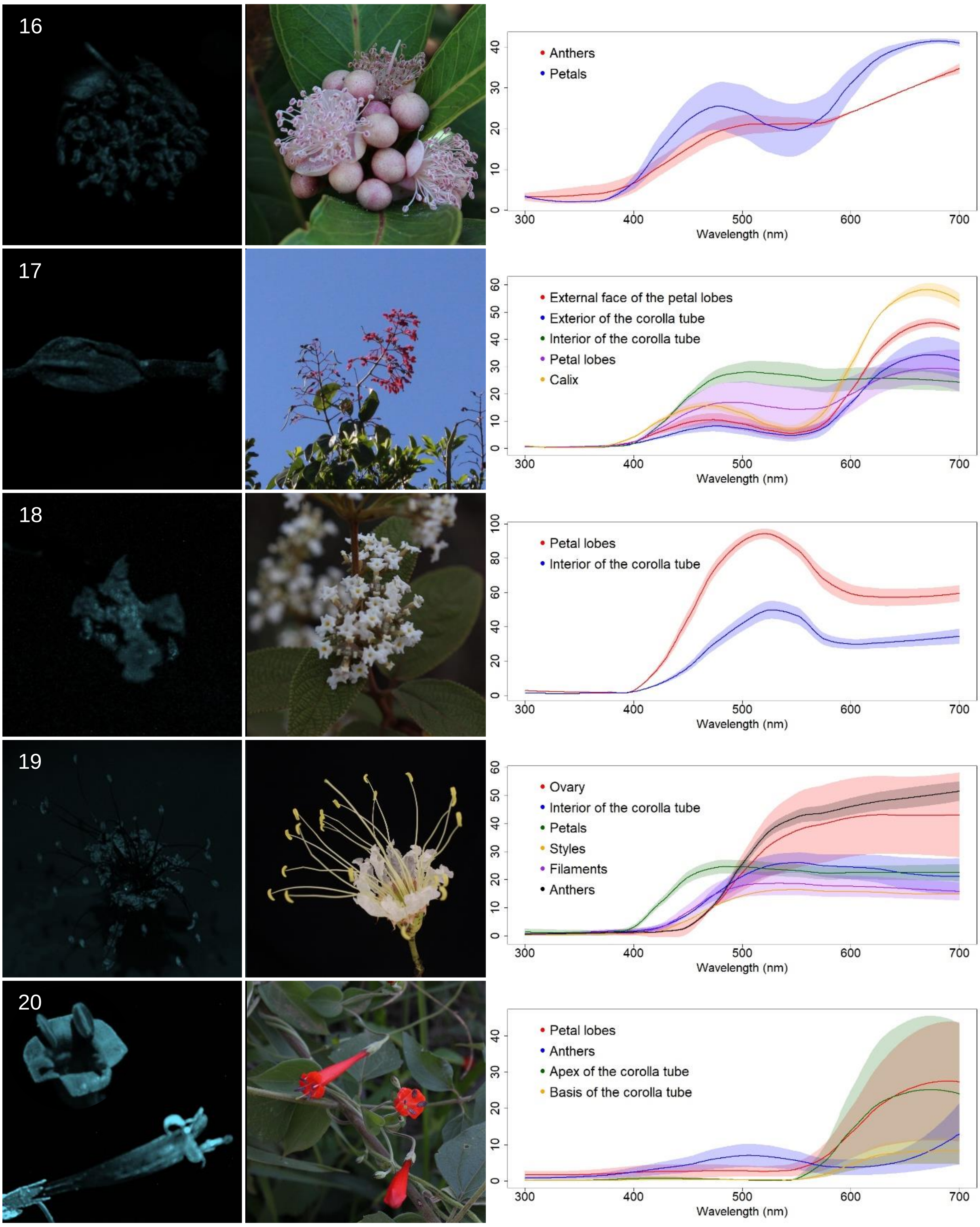
16
Anthers
Petals
Wavelength (nm)
17
External face of the petal lobes
Exterior of the corolla tube
Interior of the corolla tube
Petal lobes
Calix
Wavelength (nm)
18
Petal lobes
Interior of the corolla tube
Wavelength (nm)
19
Ovary
Interior of the corolla tube
Petals
Styles
Filaments
Anthers
Wavelength (nm)
20
Petal lobes
Anthers
Apex of the corolla tube
Basis of the corolla tube
Wavelength (nm)

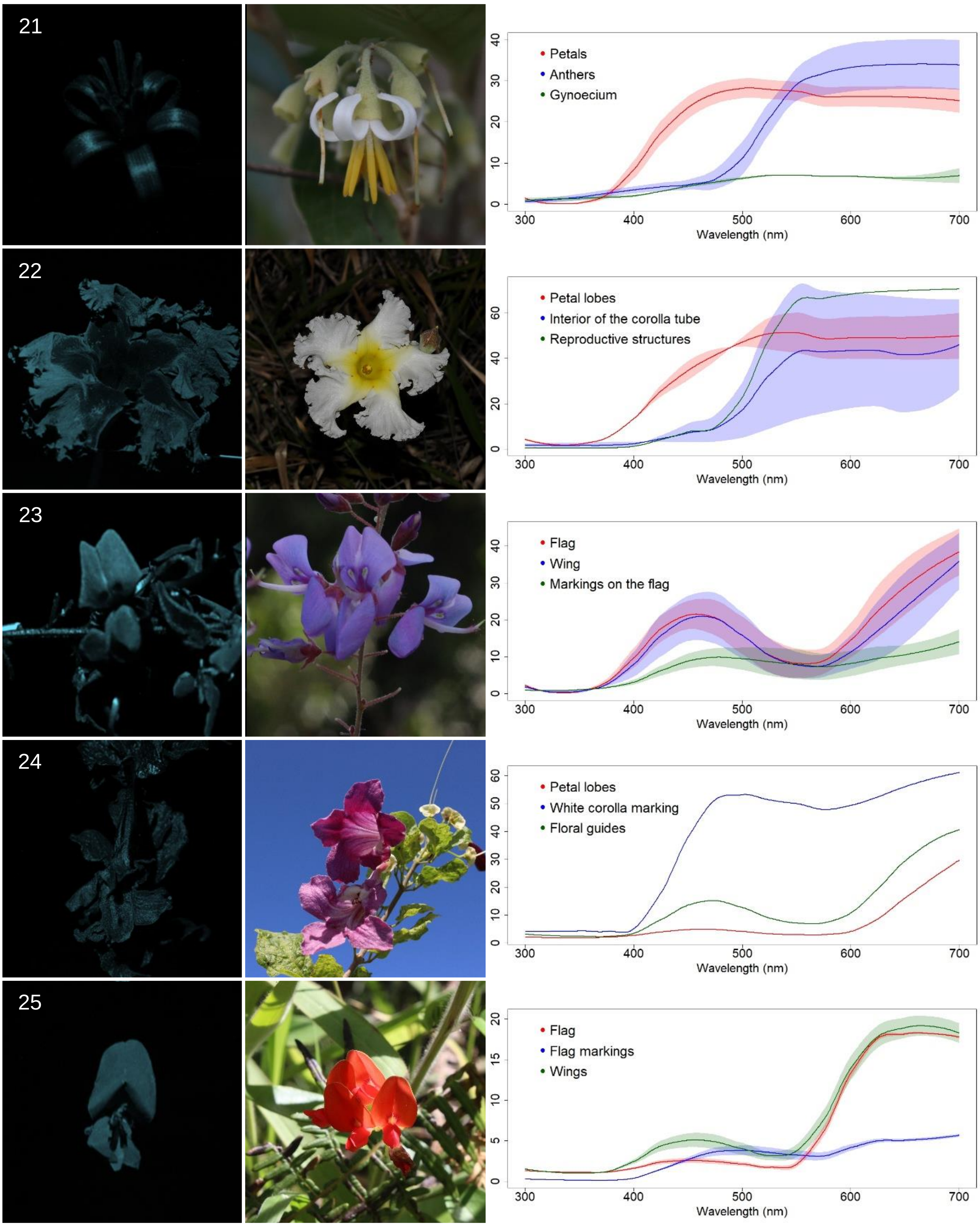
21
Petals
Anthers
Gynoecium
Wavelength (nm)
22
Petal lobes
Interior of the corolla tube
Reproductive structures
Wavelength (nm)
23
Flag
Wing
Markings on the flag
Wavelength (nm)
24
Petal lobes
White corolla marking
Floral guides
Wavelength (nm)
25
Flag
Flag markings
Wings
Wavelength (nm)

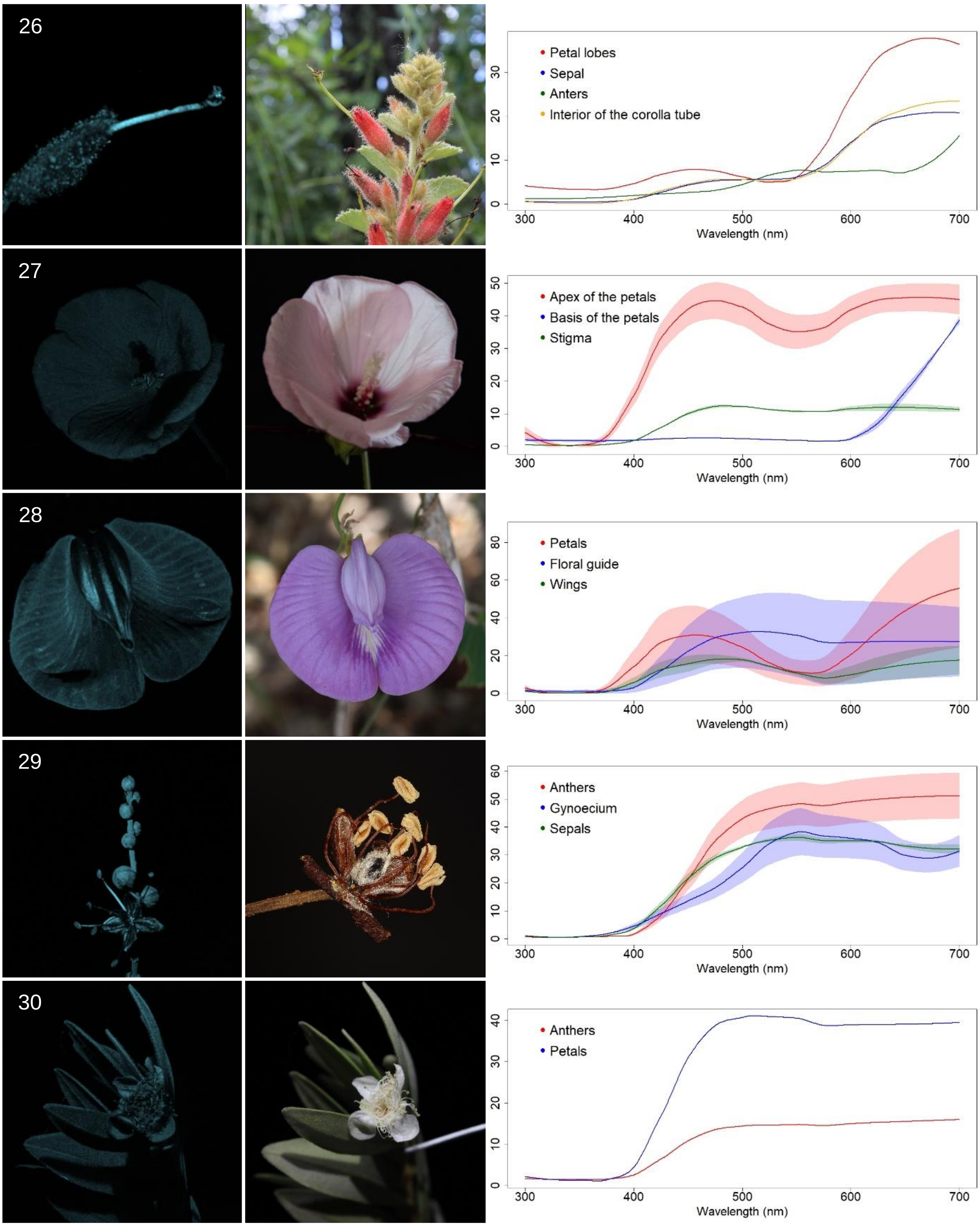

26
Petal lobes
Sepal
Anters
Interior of the corolla tube
Wavelength (nm)
27
Apex of the petals
Basis of the petals
Stigma
Wavelength (nm)
28
Petals
Floral guide
Wings
Wavelength (nm)
29
Anthers
Gynoecium
Sepals
Wavelength (nm)
30
Anthers
Petals
Wavelength (nm)

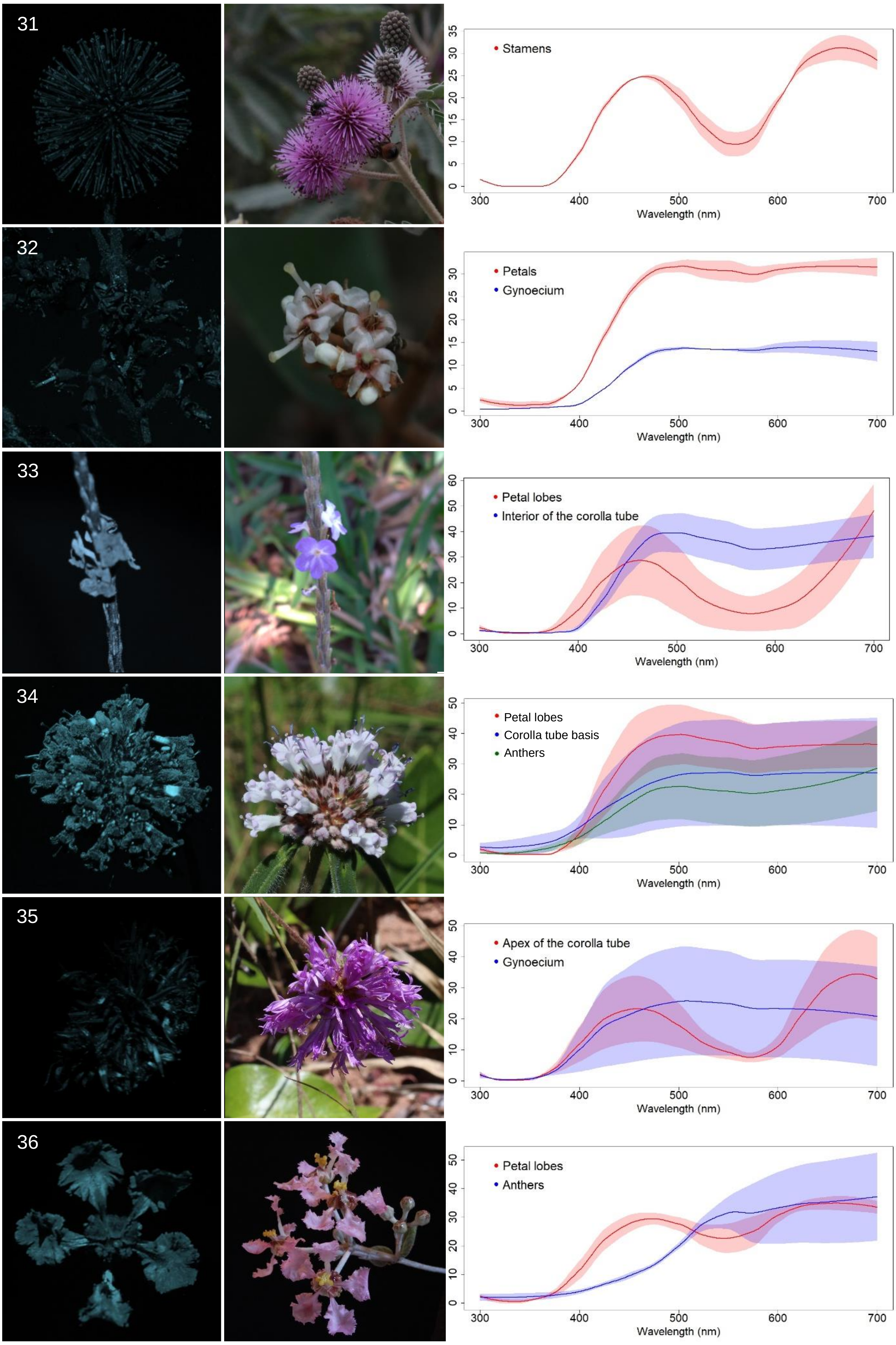
31
Stamens
Wavelength (nm)
32
Petals
Gynoecium
Wavelength (nm)
33
Petal lobes
Interior of the corolla tube
Wavelength (nm)
34
Petal lobes
Corolla tube basis
Anthers
Wavelength (nm)
35
Apex of the corolla tube
Gynoecium
Wavelength (nm)
36
Petal lobes
Anthers
Wavelength (nm)

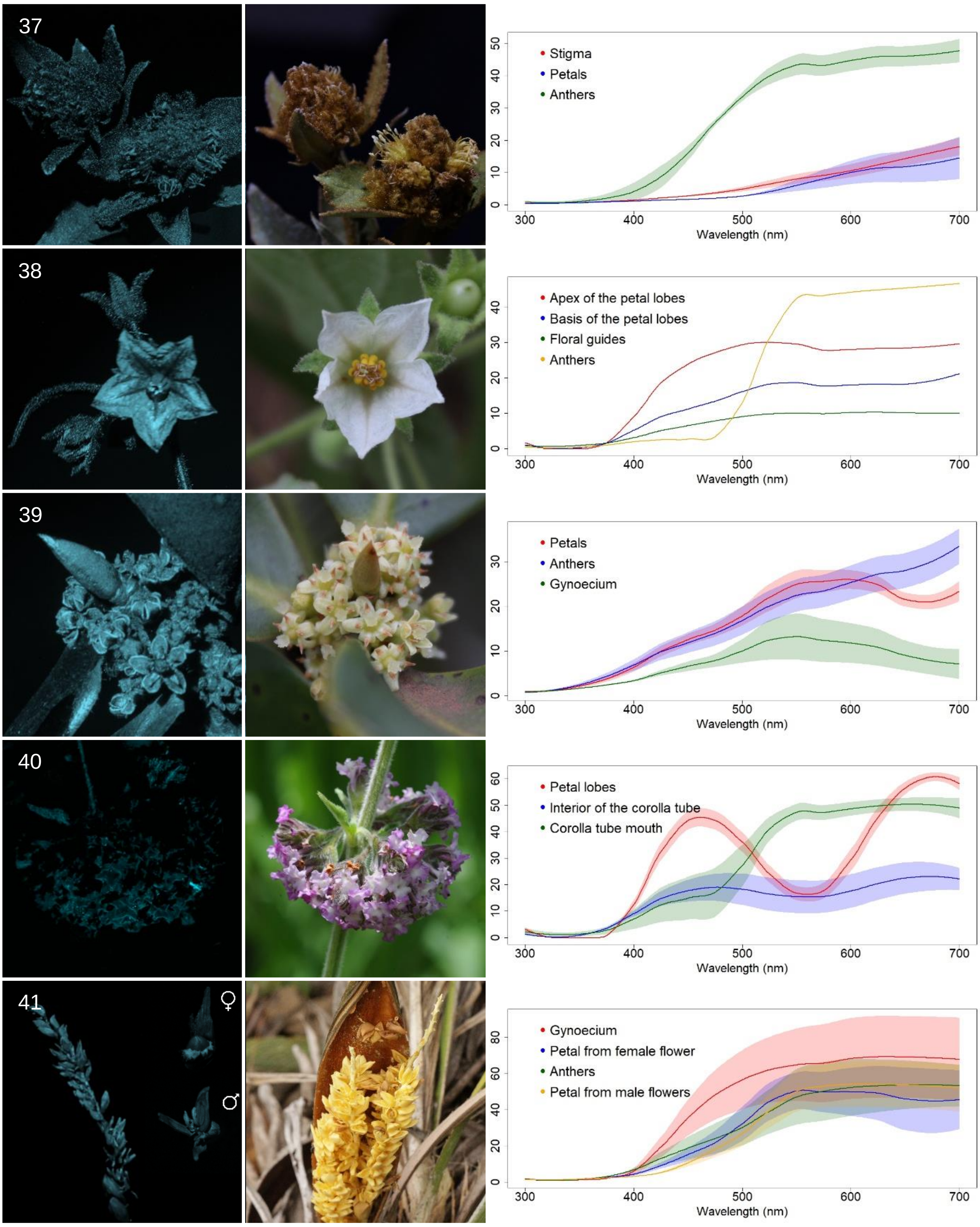
37
Stigma
Petals
Anthers
Wavelength (nm)
38
Apex of the petal lobes
Basis of the petal lobes
Floral guides
Anthers
Wavelength (nm)
39
Petals
Anthers
Gynoecium
Wavelength (nm)
40
Petal lobes
Interior of the corolla tube
Corolla tube mouth
Wavelength (nm)
41
♀
♂
Gynoecium
Petal from female flower
Anthers
Petal from male flowers
Wavelength (nm)

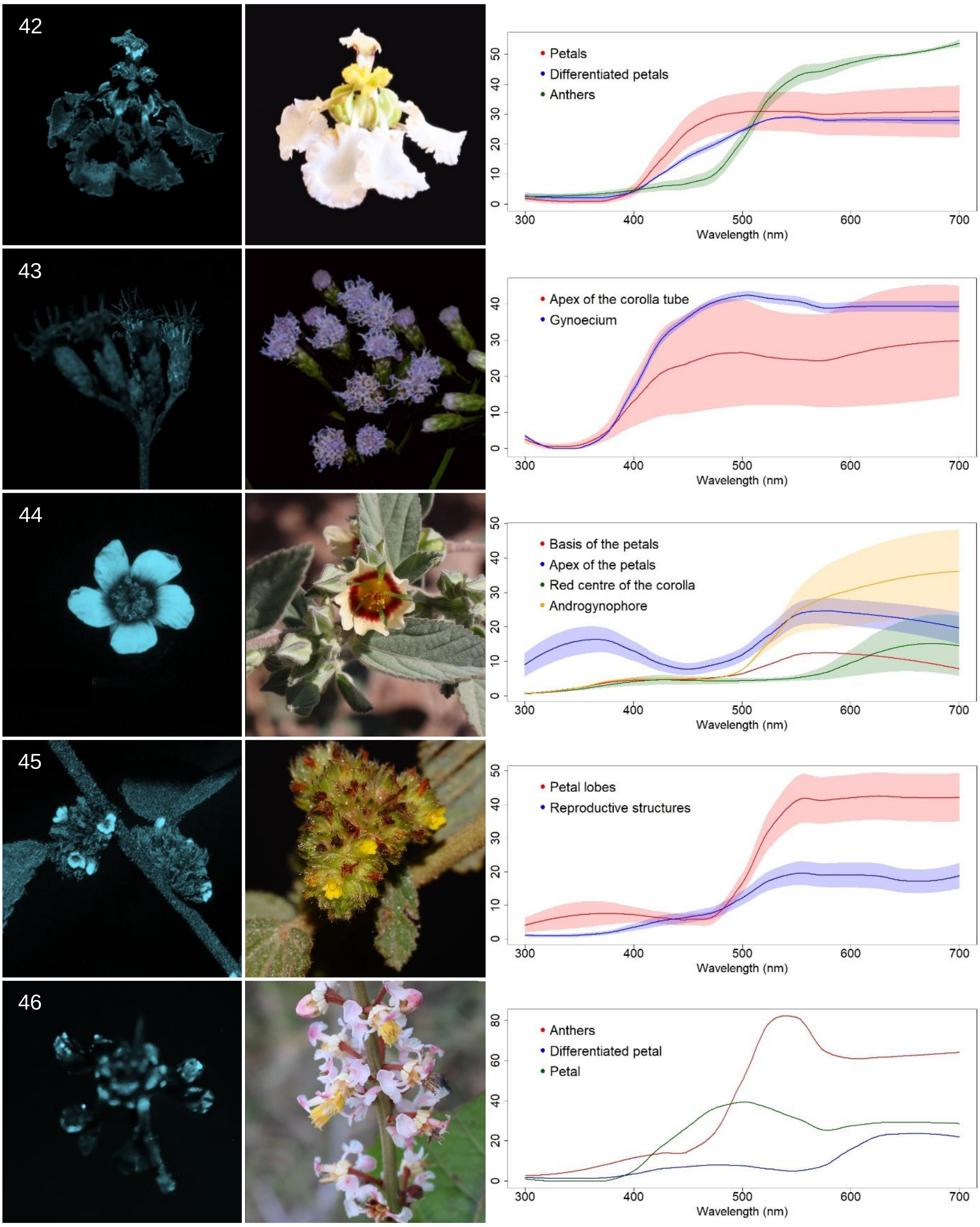
42
Petals
Differentiated petals
Anthers
Wavelength (nm)
43
Apex of the corolla tube
Gynoecium
Wavelength (nm)
44
Basis of the petals
Apex of the petals
Red centre of the corolla
Androgynophore
Wavelength (nm)
45
Petal lobes
Reproductive structures
Wavelength (nm)
46
Anthers
Differentiated petal
Petal
Wavelength (nm)

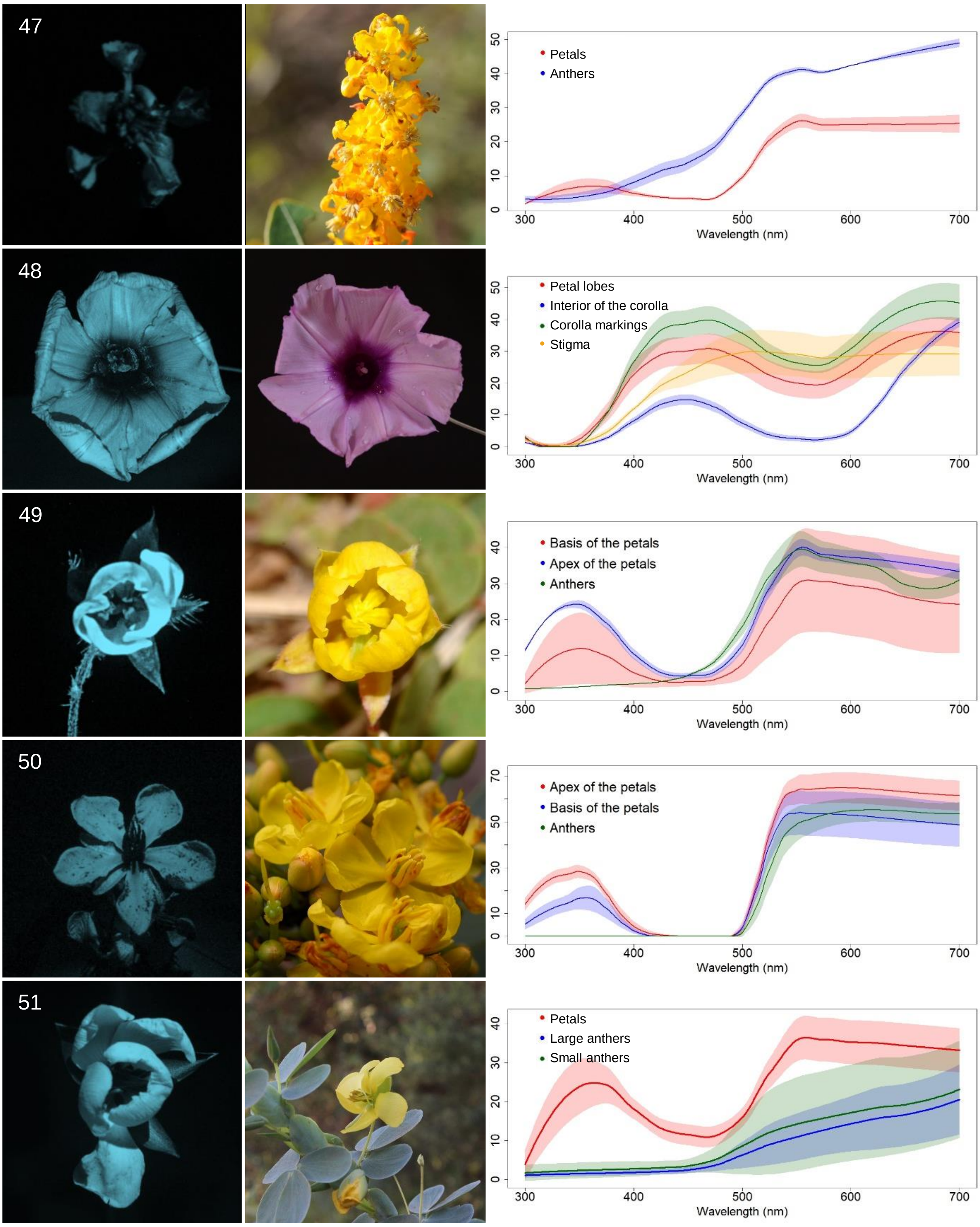

47
Petals
Anthers
Wavelength (nm)
48
Petal lobes
Interior of the corolla
Corolla markings
Stigma
Wavelength (nm)
49
Basis of the petals
Apex of the petals
Anthers
Wavelength (nm)
50
Apex of the petals
Basis of the petals
Anthers
Wavelength (nm)
51
Petals
Large anthers
Small anthers
Wavelength (nm)

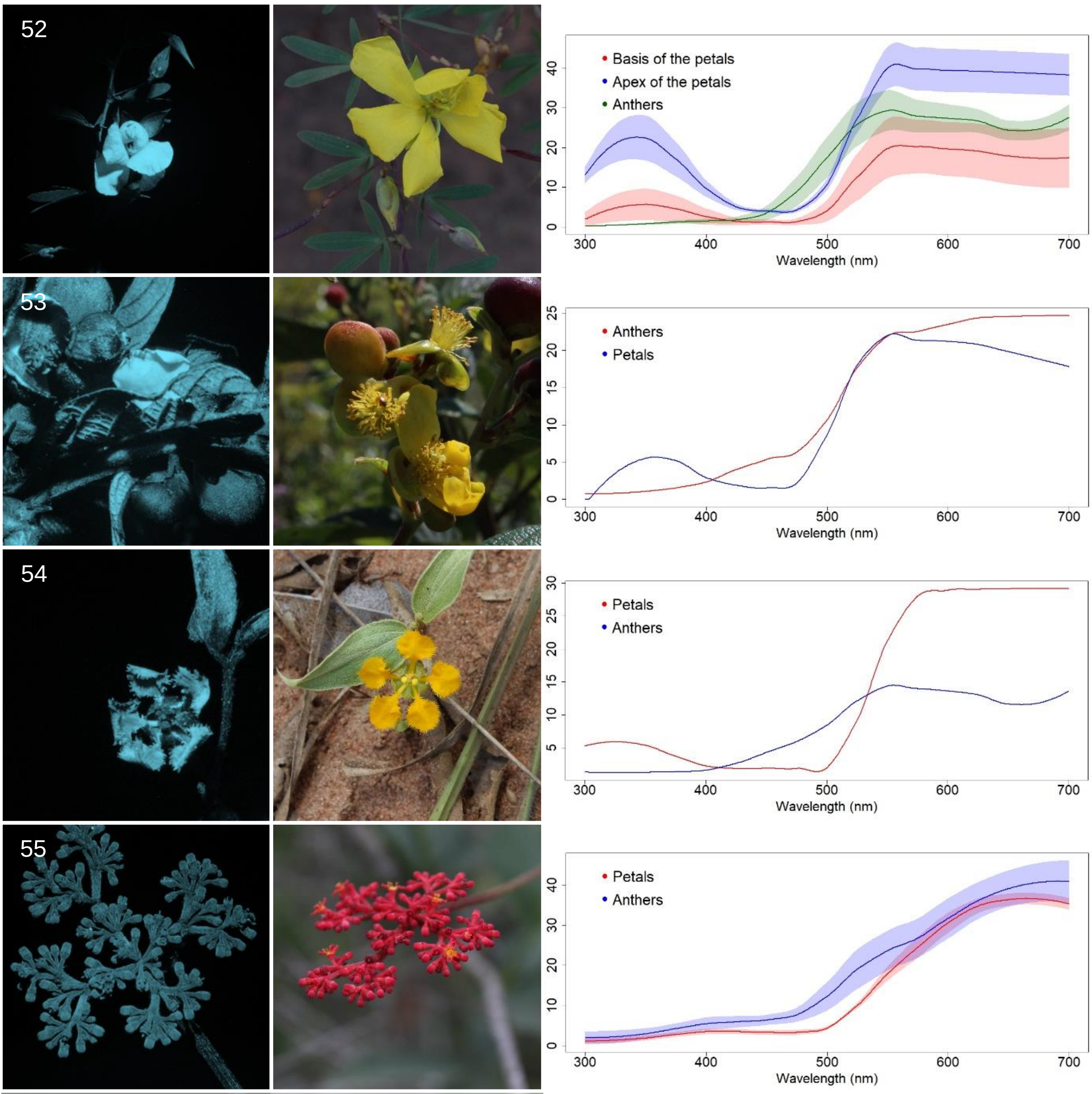
52
Basis of the petals
Apex of the petals
Anthers
0 10 20 30 40
300 400 500 600 700
Wavelength (nm)
53
Anthers
Petals
0 5 10 15 20 25
300 400 500 600 700
Wavelength (nm)
54
Petals
Anthers
5 10 15 20 25 30
300 400 500 600 700
Wavelength (nm)
55
Petals
Anthers
0 10 20 30 40
300 400 500 600 700
Wavelength (nm)
