# Supplementary material for: Floral UV Features of Plant Species From a Neotropical Savanna
Source: Front Plant Sci. 2021 May 7;12:618028. doi: 10.3389/fpls.2021.618028 (PMC8137824; doi:10.3389/fpls.2021.618028)
Supplement: Supplementary file 2 [file Data_Sheet_2.pdf]

Supplementary Material

Supplementary Table 1. Percentage of plant species showing each pollination system in Neotropical savanna communities

Pollination systems	Plant species (%)		
	Gottsberger and Silberbauer-Gottsberger, 2006	Tunes et al., 2017	This study
Generalist-pollination	42.5	41.7	14.8
Bee-pollination	43.7	38.3	55.5
Beetle-pollination	3.1	5.0	2.5
Hummingbird-pollination	1.9	5.0	9.9
Butterfly-pollination	1.1	6.7	9.9
Moth-pollination	2.3	0.0	0.0
Hawkmoth-pollination	2.7	1.7	2.5
Fly-pollination	1.5	0.0	1.2
Bat-pollination	1.1	1.7	3.7
**Total**	100.0	100.0	100.0

Supplementary Table 2. List of the 80 sampled plant species from Neotropical savanna with their respective UV categories**,** pollination system, types of resources collected by pollinators and attraction units (based on Ramirez et al., 1990). R = UV-reflecting, CM = contrasting markings in the corolla oriented towards floral resources, CR = contrasting reproductive structures, A = UV-absorbing, BE = bullseye, P = Pollen, T = Tissue, N = Nectar, O = Oil.

Species	UV-categories	Pollination system	Floral resources	Attraction unit	Non-UV pattern
**Amaranthaceae**					
Gomphrena macrocephala A.St.-Hil.	A	Generalist	P, N	Inflorescence	Present
**Annonaceae**					
Annona coriacea Mart.	CR	Cantharophily [a, b]	P, T [c]	Flower	Present
**Apocynaceae**					
Mandevilla longiflora (Desf.) Pichon	A	Sphingophily [d]	N [d]	Flower	Present
Mandevilla pohliana (Stadelm.) A.H.Gentry	A	Melittophilous [a]	N [c]	Flower	Present
Oxypetalum appendiculatum Mart.	BE	Melittophilous [e]	N [e]	Flower	Present
Temnadenia violacea (Vell.) Miers	CM	Melittophilous [a]	N [c]	Flower	Present
**Arecaceae**					
Syagrus loefgrenii Glassman	A	Cantharophily [f]	P, N [f]	Inflorescence	Absent
**Bignoniaceae**					
Adenocalymma peregrinum (Miers) L.G.Lohmann	R	Melittophilous [a]	N [c]	Flower	Absent
Amphilophium mansoanum (DC.) L.G.Lohmann	A	Melittophilous [g, h]	N [g, h]	Flower	Present
Cuspidaria sp.	BE	Melittophilous [e]	N [e]	Flower	Present
Fridericia samydoides (Cham.) L.G.Lohmann	A	Melittophilous [i]	N [i]	Flower	Present
Fridericia speciosa Mart.	A	Melittophilous [i]	N [i]	Flower	Present
Jacaranda caroba (Vell.) DC.	R	Melittophilous [a]	N [c]	Flower	Present
Jacaranda rufa Silva Manso	A	Melittophilous [a]	N [c]	Flower	Present
Pyrostegia venusta (Ker Gawl.) Miers	A	Ornithophilous [a]	N [c]	Flower	Absent
Zeyheria montana Mart	R	Ornithophilous [a]	N [c]	Flower	Present

**Caryocaraceae**					
Caryocar brasiliense A.St.-Hil.	A	Chiropterophilous [a]	N [c]	Flower	Absent
**Compositae**					
Bidens gardneri Baker	A	Melittophilous [a]	P, N [c]	Inflorescence	Present
Calea triantha (Vell.) Pruski	A	Generalist [e]	N [e]	Inflorescence	Absent
Chromolaena laevigata (Lam.) R.M.King & H.Rob.	A	Generalist [a]	P, N [c]	Inflorescence	Absent
Emilia fosbergii Nicolson	A	Generalist [e]	N [e]	Inflorescence	Absent
Lessingianthus sp.	A	Generalist [e]	N [e]	Inflorescence	Present
Tridax procumbens (L.) L.	A	Psychophilous [j]	P, N [j]	Inflorescence	Present
**Convolvulaceae**					
Ipomoea delphinioides Choisy	CR	Melittophilous [e]	N [e]	Flower	Present
Merremia digitata (Spreng.) Hallier f.	A	Melittophilous [a]	N [c]	Flower	Absent
**Dilleniaceae**					
Davilla elliptica A.St.-Hil.	CR	Generalist [a]	P [c]	Flower	Absent
**Euphorbiaceae**					
Croton campestris A.St.-Hil.	A	Melittophilous [e]	P, N [e]	Flower	Present
**Lamiaceae**					
Salvia minarum Briq.	CM	Melittophilous [e]	N [e]	Flower	Present
**Leguminosae**					
Bauhinia rufa (Bong.) Steud.	R	Chiropterophilous [a]	N [c]	Flower	Present
Betencourtia scarlatina (Mart. ex Benth,) L.P.Queiroz	A	Ornithophilous [k]	N [k]	Flower	Present
Calliandra dysantha Benth.	A	Ornithophilous [l]	N [l]	Inflorescence	Absent
Centrosema angustifolium (Kunth) Benth.	A	Melittophilous [e]	N [e]	Flower	Present
Chamaecrista desvauxii var. *desvauxii*	CR	Melittophilous [m]	P [m]	Flower	Absent
Chamaecrista ramosa (Vogel) H.S.Irwin & Barneby	CR	Melittophilous [e]	P [e]	Flower	Absent
Chamaecrista rotundifolia (Pers.) Greene	CR	Melittophilous [a]	P [c]	Flower	Absent
Copaifera langsdorfii Desf.	A	Melittophilous [a]	N [c]	Flower	Absent

Crotalaria micans Link	CM	Melittophilous [n]	N [n]	Flower	Present
Desmodium subsecundum Vogel	A	Melittophilous [e]	N [e]	Flower	Present
Mimosa lanata Benth.	A	Melittophilous [e]	P [e]	Inflorescence	Absent
Senna rugosa (G.Don) H.S.Irwin & Barneby	CR	Melittophilous [a]	P [c]	Flower	Present
Stylosanthes guianensis (Aubl.) Sw.	R	Melittophilous [a]	N [c]	Flower	Present
**Lentibulariaceae**					
Utricularia triloba Benj.	R	Melittophilous [e]	N [e]	Flower	Present
**Lythraceae**					
Lafoensia pacari A.St.-Hil..	A	Chiropterophilous [o]	N [o]	Flower	Present
**Malpighiaceae**					
Aspicarpa pulchella (Griseb.) O'Donell & Lourteig	CR	Melittophilous [p]	O, P [p]	Flower	Absent
Banisteriopsis argyrophylla (A.Juss.) B.Gates	A	Melittophilous [q]	O [q]	Flower	Present
Banisteriopsis campestris (A.Juss.) Little	A	Melittophilous [a]	O, P [c]	Flower	Present
Byrsonima coccolobifolia Kunth	CR	Melittophilous [a]	O [c]	Flower	Present
Byrsonima intermedia A.Juss.	CR	Melittophilous [a]	O, P [c]	Flower	Absent
Janusia guaranitica (A.St.-Hil.) A.Juss.	CR	Melittophilous [r]	O, P [r]	Flower	Absent
**Malvaceae**					
Helicteres sacarolha A.St.-Hil., Juss. & Cambess.	A	Ornithophilous [s]	N [s]	Flower	Present
Pavonia sp.	A	Melittophilous [e]	P, N [e]	Flower	Present
Peltaea polymorpha (A. St.-Hil.) Krapov. & Cristóbal	BE	Melittophilous [e]	N [e]	Flower	Present
Sida cf. *urens*	BE	Generalist [a]	P, N [c]	Flower	Present
Waltheria indica L.	BE	Generalist [a]	N [c]	Intermediate	Absent
**Melastomataceae**					
Miconia albicans (Sw.) Triana	A	Melittophilous [t]	P [t]	Flower	Absent
Miconia ligustroides (DC.) Naudin	A	Melittophilous [a]	P [c]	Flower	Absent
Pleroma stenocarpa (DC.) Cogn.	CR	Melittophilous [e]	P [e]	Flower	Present
**Myrsinaceae**					

Myrsine guianensis (Aubl.) Kuntze	A	Generalist[a]	N[c]	Flower	Present
**Myrtaceae**					
Eugenia sp.	A	Melittophilous[e]	P[e]	Flower	Absent
Psidium sp.	A	Melittophilous[u, v]	P[u, v]	Flower	Present
**Ochnaceae**					
Ouratea spectabilis (Mart. ex Engl.) Engl.	CR	Melittophilous[a]	P[c]	Flower	Absent
**Onagraceae**					
Ludwigia nervosa (Poir.) H.Hara	BE	Melittophilous[w]	P, N[w]	Flower	Absent
**Orchidaceae**					
Epistephium sclerophyllum Lindl.	CM	Melittophilous[a]	N[c]	Flower	Present
**Passifloraceae**					
Passiflora cincinnata Mast.	CR	Melittophilous[x]	N[x]	Flower	Present
**Rubiaceae**					
Borreria tenella (Kunth) Cham. & Schltdl.	A	Generalist[a]	N[c]	Inflorescence	Present
Manettia cordifolia Mart.	A	Ornithophilous[y]	N[y]	Flower	Present
Palicourea rigida Kunth	A	Ornithophilous[a]	N[c]	Flower	Absent
Spermacoce poaya A.St.-Hil.	R	Melittophilous[a]	N[c]	Flower	Absent
Tocoyena formosa (Cham. & Schltdl.) K.Schum.	A	Sphingophilous[a]	N[c]	Flower	Absent
**Solanaceae**					
Solanum sp.	A	Melittophilous[e]	P[e]	Flower	Present
**Styracaceae**					
Styrax camporum Pohl	A	Melittophilous[a]	N[c]	Flower	Present
**Turneraceae**					
Piriqueta aurea (Cambess.) Urb.	BE	Melittophilous[e]	P, N[e]	Flower	Present
**Verbenaceae**					
Lantana camara L.	A	Psychophilous[a]	N[c]	Intermediate	Present
Lippia alba (Mill.) N.E.Br. ex Britton & P.Wilson	A	Psychophilous[e]	N[e]	Intermediate	Present

	Lippia lasiocalycina Cham.	A	Psychophilous [a]	N [c]	Intermediate	Present
	Lippia lupulina Cham.	A	Psychophilous [a]	N [c]	Intermediate	Present
	Lippia origanoides Kunth	A	Psychophilous [a]	N [c]	Inflorescence	Absent
	Lippia stachyoides Cham.	A	Psychophilous [a]	N [c]	Intermediate	Present
	Stachytarpheta cayennensis (Rich.) Vahl	A	Melittophilous [e]	N [e]	Flower	Present
**Vitaceae**						
	Cissus erosa Rich.	CR	Myophilous [a]	N [c]	Flower	Absent

References

[a] Gottsberger, G., and Silberbauer-Gottsberger, I. (2006). Life in the Cerrado: pollination and seed dispersal (Vol. 2). Ulm: Reta Verlag.

[b] Costa, M. S., Silva, R. J., Paulino-Neto, H. F., and Pereira, M. J. B. (2017). Beetle pollination and flowering rhythm of *Annona coriacea* Mart.(Annonaceae) in Brazilian cerrado: Behavioral features of its principal pollinators. PLoS One, 12(2), e0171092.

[c] Resources collected by pollinators adapted from Gottsberger and Silberbauer-Gottsberger, 2006.

[d] Moré, M., Sérsic, A. N., and Cocucci, A. A. (2007). Restriction of pollinator assemblage through flower length and width in three long-tongued hawkmoth–pollinated species of *Mandevilla* (Apocynaceae, Apocynoideae) 1. Annals of the Missouri Botanical Garden, 94(2), 485-504.

[e] Personal classification based on Faegri and Van der Pijl, 1979 and Rosas-Guerrero et al., 2014. Faegri, K., and Van der Pijl, L. (1979). The Principles of Pollination Ecology. 3rd ed. Oxford: Pergamom Press. Rosas-Guerrero, V., Aguilar, R., Martén-Rodríguez, S., Ashworth, L., Lopezaraiza-Mikel, M., Bastida, J. M., and Quesada, M. (2014). A quantitative review of pollination syndromes: do floral traits predict effective pollinators?. Ecol. Lett. 17(3), 388-400.

[f] Silberbauer-Gottsberger, I. (1990). Pollination and evolution in palms. Phyton, 30(2), 213-233.

[g] Personal field observation.

[h] Marchi, P., and Alves-dos-Santos, I. (2013). The bees of the genus *Xylocopa* Latreille (Xylocopini, Apidae) of São Paulo State, Brazil. Biota Neotropica, 13(2), 249-269.

[i] See references in Ono, E. R., Valentin-Silva, A., and Guimarães, E. (2020). Spatial and temporal distribution of floral resources used by pollinators in a semi-deciduous seasonal forest. The International Journal of Plant Reproductive Biology, 12(1), 11-24.

[j] Thakur, M. S., and Mattu, V. K. (2010). The role of butterfly as flower visitors and pollinators in Shiwalik hills of western Himalayas. Asian J. Exp. Biol. Sci. 4, 822-825.

[k] Sazima, I., Buzato, S., and Sazima, M. (1996). An assemblage of hummingbird-pollinated flowers in a montane forest in southeastern Brazil. Bot. Acta. 109(2), 149-160.

[l] Tunes, P. T. (2017). Influência da florivoria sobre a polinização de espécies ornitófilas. [Master’s thesis]. [Botucatu (SP)]: São Paulo State University.

[m] Nogueira, A., Valadão-Mendes, L. B., El Ottra, J. H., Guimarães, E., Cardoso-Gustavson, P., Quinalha, M. M., ... and Rando, J. G. (2018). Relationship of floral morphology and development with the pattern of bee visitation in a species with pollen-flowers, *Chamaecrista desvauxii* (Fabaceae). Botanical Journal of the Linnean Society, 187(1), 137-156.

[n] Gottsberger, G.; Etcheverry, A. V., Protomastro, J. J., and Westerkamp, C. (2003). Delayed autonomous self-pollination in the colonizer *Crotalaria micans* (Fabaceae: Papilionoideae): structural and functional aspects. Plant Systematics and Evolution, 239(1), 15-28.

[o] Sazima M, and Sazima I. (1975). Quiropterofilia em *Lafoensia pacari* St. Hil. (Lythraceae), na Serra do Cipó, Minas Gerais. Ciência e Cultura 27: 405–416.

[p] Tunes, P., Alves, V. N., Valentin-Silva, A., Batalha, M. A., and Guimarães, E. (2017). Does fire affect the temporal pattern of trophic resource supply to pollinators and seed-dispersing frugivores in a Brazilian savanna community?. Plant Ecol. 218(3), 345-357.

[q] Aidar, I. F., Bartelli, B. F., and Nogueira-Ferreira, F. H. (2015). Network of bee-plant interactions and recognition of key species in semideciduous forest. Sociobiology, 62(4), 583-592.

[r] Gonçalves, L., Silva, C. I., and Buschini, M. L. T. (2012). Collection of pollen grains by Centris (Hemisiella) tarsata Smith (Apidae: Centridini): is C. tarsata an oligolectic or polylectic species. Zoological Studies, 51(2), 195-203.

[s] Franceschinelli, E. V. (2005). The pollination biology of two species of *Helicteres* (Malvaceae) with different mechanisms of pollen deposition. Flora-Morphology, Distribution, Functional Ecology of Plants, 200(1), 65-73.

[t] Mesquita-Neto, J. N., Blüthgen, N., and Schlindwein, C. (2018). Flowers with poricidal anthers and their complex interaction networks—Disentangling legitimate pollinators and illegitimate visitors. Functional Ecology, 32(10), 2321-2332.

[u] Anita, M., Sivaram, V., and Jayaramappa, K. V. (2012). Influence of bee attractants on pollination and yield parameters in Guava (*Psidium guajava* L.). Int. J. Plant Reproductive Biology, 4(1).

[v] Hansen, K., Sritongchuay, T., Bumrungsri, S., Simmons, B. I., Strange, N., and Dalsgaard, B. (2020). Landscape-level effects of forest on pollinators and fruit set of guava (*Psidium guajava* L.) in orchards across southern Thailand. Diversity, 12(6), 259.

[w] Ferreira E. A., Boff, S., Verza, S. S., and Mussury, R. M. (2021). Bioecological and Behavioral Interaction between Pollinating Bees and the Pioneer Shrub *Ludwigia nervosa* in Degraded Area Suggests an Exotic Bee as Its Major Pollinator. *Biology*, *10*(2), 114.

[x] Malerbo Souza, D. T. (2011). La biodiversidad de los polinizadores en *Passiflora cincinnata* Mast.(Passifloraceae) en Ribeirão Preto, Brasil. Zootecnia Tropical, 29(1), 17-27.

[y] Consolaro, H., Silva, E. B. D., and Oliveira, P. E. D. (2005). Variação floral e biologia reprodutiva de *Manettia cordifolia* Mart.(Rubiaceae). Brazilian Journal of Botany, 28(1), 85-94.

Supplementary Table 3. Number (and percentage) of species in each UV category with each pollination system. R = UV-reflecting, A = UV-absorbing, BE = bullseye, CM = contrasting markings on the corolla oriented towards floral resources, CR = contrasting reproductive structures

**Pollination systems**	**UV-categories**				
	**R**	**A**	**BE**	**CM**	**CR**
Bee-pollination	4 (66.6)	19 (40.4)	5 (71.4)	5 (100.0)	11 (80.0)
Generalist-pollination	0 (0.0)	9 (17.0)	2 (28.6)	0 (0.0)	1 (6.7)
Hummingbird-pollination	1 (16.7)	7 (14.9)	0 (0.0)	0 (0.0)	0 (0.0)
Beetle-pollination	0 (0.0)	1 (2.1)	0 (0.0)	0 (0.0)	1 (6.7)
Hawkmoth-pollination	0 (0.0)	2 (4.3)	0 (0.0)	0 (0.0)	0 (0.0)
Fly-pollination	0 (0.0)	0 (0.0)	0 (0.0)	0 (0.0)	1 (6.6)
Butterfly-pollination	0 (0.0)	8 (17.0)	0 (0.0)	0 (0.0)	0 (0.0)
Bat-pollination	1 (16.7)	2 (4.3)	0 (0.0)	0 (0.0)	0 (0.0)
**Total**	6 (100.0)	48 (100.0)	7 (100.0)	5 (100.0)	14 (100.0)

Supplementary Table 4. Post-hoc pairwise comparison performed after asymptotic generalized Pearson chi-squared test of the proportion of pollination modes within each UV category. Similar letters indicate that the UV category presented similar proportion of pollination modes. In bold are the p-values considered significant ($p < 0.05$). R = UV-reflecting, A = UV-absorbing, BE = bullseye CM = contrasting markings on the corolla oriented towards floral resources, CR = contrasting reproductive structures

Pairwise comparison	p-value
R^{AB} - CM^{AB}	0.2240
R^{AB} - CR^{A}	0.3160
R^{AB} - A^{B}	0.6200
R^{AB} - BE^{AB}	0.2440
CM^{AB} - CR^{A}	0.3500
CM^{AB} - A^{B}	0.0686
CM^{AB} - BE^{AB}	0.1900
CR^{A} - A^{B}	**0.0359**
CR^{A} - BE^{AB}	0.5660
A^{B} - BE^{AB}	0.0649

Supplementary Table 5. Number (and percentage) of species presenting each UV category with each type of floral resource. R = UV-reflecting, A = UV-absorbing, BE = bullseye, CM = contrasting markings on the corolla oriented towards floral resources, CR = contrasting reproductive structures

**Floral resources**	**UV categories**				
	**R**	**A**	**BE**	**CM**	**CR**
Nectar	6 (100)	39 (70.9)	7 (70.0)	5 (100.0)	3 (15.8)
Pollen	0 (0.0)	14 (25.5)	3 (30.0)	0 (0.0)	11 (57.9)
Oil	0 (0.0)	2 (3.6)	0 (0.0)	0 (0.0)	4 (21.0)
Tissue	0 (0.0)	0 (0.0)	0 (0.0)	0 (0.0)	1 (5.3)
Total	6 (100.0)	55 (100.0)	10 (100.0)	5 (100.0)	19 (100.0)

Supplementary Table 6. Post-hoc pairwise comparison performed after asymptotic generalized Pearson chi-squared test of the proportion of floral resources within each UV category. Similar letters indicate that the UV categories presented similar proportion of floral resources. In bold are the p-values considered significant ($p < 0.05$). R = UV-reflecting, A = UV-absorbing, BE = bullseye CM = contrasting markings on the corolla oriented towards floral resources, CR = contrasting reproductive structures

Pairwise comparison	p-value
R^A - CM^A	1
R^A – CR^B	**$2.44*10^{-3}$**
R^A – A^A	0.146
R^A - BE^A	0.137
CM^A – CR^B	**$4.56*10^{-3}$**
CM^A - A^A	0.183
CM^A - BE^A	0.171
CR^B - A^A	**$8.51*10^{-6}$**
CR^B - BE^A	**$4.64*10^{-3}$**
A^A - BE^A	0.881

Supplementary Table 7. Number (and percentage) of species presenting each UV category with each type of attraction unit. R = UV-reflecting, A = UV-absorbing, BE = bullseye, CM = contrasting markings on the corolla oriented towards floral resources, CR = contrasting reproductive structures

**Attraction unit**	**UV categories**				
	**R**	**A**	**BE**	**CM**	**CR**
Flower	5 (83.3)	29 (61.7)	6 (85.7)	5 (100.0)	15 (100.0)
Inflorescence	1 (16.7)	6 (12.8)	1 (14.3)	0 (0.0)	0 (0.0)
Intermediate	0 (0.0)	12(25.5)	0 (0.0)	0 (0.0)	0 (0.0)
Total	6 (100.0)	47 (100.0)	7 (100.0)	5 (100.0)	15 (100.0)

Supplementary Table 8. Post-hoc pairwise comparison performed after asymptotic generalized Pearson chi-squared test of the proportion of attraction unit types within each UV category. Similar letters indicate that the UV categories presented similar proportion of floral resources. In bold are the p-values considered significant ($p < 0.05$). R = UV-reflecting, A = UV-absorbing, BE = bullseye CM = contrasting markings on the corolla oriented towards floral resources, CR = contrasting reproductive structures

Pairwise comparison	p-value
R^{AB} - CM^{AB}	0.33800
R^{AB} - CR^{A}	0.10500
R^{AB} - A^{B}	0.19200
R^{AB} - BE^{AB}	0.90600
CM^{AB} - CR^{A}	1
CM^{AB} - A^{B}	0.10600
CM^{AB} - BE^{AB}	0.37700
CR^{A} - A^{B}	**0.00699**
CR^{A} - BE^{AB}	0.13400
A^{B} - BE^{AB}	0.14100

Supplementary Table 9. Number (and percentage) of species presenting each UV category with and without non-UV colour patterns. R = UV-reflecting, A = UV-absorbing, BE = bullseye, CM = contrasting markings on the corolla oriented towards floral resources, CR = contrasting reproductive structures

Presence/ absence of non-UV colour patterns	UV categories				
	R	A	BE	CM	CR
Presence of non-UV colour pattern	3 (50.0)	30 (63.8)	5 (71.4)	5 (100.0)	6 (40.0)
Absence of non-UV colour pattern	3 (50.0)	17 (36.2)	2 (28.6)	0 (0.0)	9 (60.0)
Total	6 (100.0)	47 (100.0)	7 (100.0)	5 (100.0)	15 (100.0)
